# Two-Dimensional Near-Atom-Thickness Materials for Emerging Neuromorphic Devices and Applications

**DOI:** 10.1016/j.isci.2020.101676

**Published:** 2020-10-13

**Authors:** Tae-Jun Ko, Hao Li, Sohrab Alex Mofid, Changhyeon Yoo, Emmanuel Okogbue, Sang Sub Han, Mashiyat Sumaiya Shawkat, Adithi Krishnaprasad, Molla Manjurul Islam, Durjoy Dev, Yongjun Shin, Kyu Hwan Oh, Gwan-Hyoung Lee, Tania Roy, Yeonwoong Jung

**Affiliations:** 1 NanoScience Technology Center, University of Central Florida, Orlando, FL 32826, USA; 2 Department of Electrical and Computer Engineering, University of Central Florida, Orlando, FL 32816, USA; 3 Department of Materials Science and Engineering, Seoul National University, Seoul, 08826, South Korea; 4 Department of Physics, University of Central Florida, Orlando, FL 32816, USA; 5 Research Institute of Advanced Materials (RIAM), Seoul National University, Seoul, 08826, South Korea; 6 Institute of Engineering Research, Seoul National University, Seoul, 08826, South Korea; 7 Institute of Applied Physics, Seoul National University, Seoul, 08826, South Korea; 8 Department of Materials Science and Engineering, University of Central Florida, Orlando, FL 32816, USA

**Keywords:** Electronic Engineering, Materials Science, Electrical Property

## Abstract

Two-dimensional (2D) layered materials and their heterostructures have recently been recognized as promising building blocks for futuristic brain-like neuromorphic computing devices. They exhibit unique properties such as near-atomic thickness, dangling-bond-free surfaces, high mechanical robustness, and electrical/optical tunability. Such attributes unattainable with traditional electronic materials are particularly promising for high-performance artificial neurons and synapses, enabling energy-efficient operation, high integration density, and excellent scalability. In this review, diverse 2D materials explored for neuromorphic applications, including graphene, transition metal dichalcogenides, hexagonal boron nitride, and black phosphorous, are comprehensively overviewed. Their promise for neuromorphic applications are fully discussed in terms of material property suitability and device operation principles. Furthermore, up-to-date demonstrations of neuromorphic devices based on 2D materials or their heterostructures are presented. Lastly, the challenges associated with the successful implementation of 2D materials into large-scale devices and their material quality control will be outlined along with the future prospect of these emergent materials.

## Graphical Abstract

**Figure:**
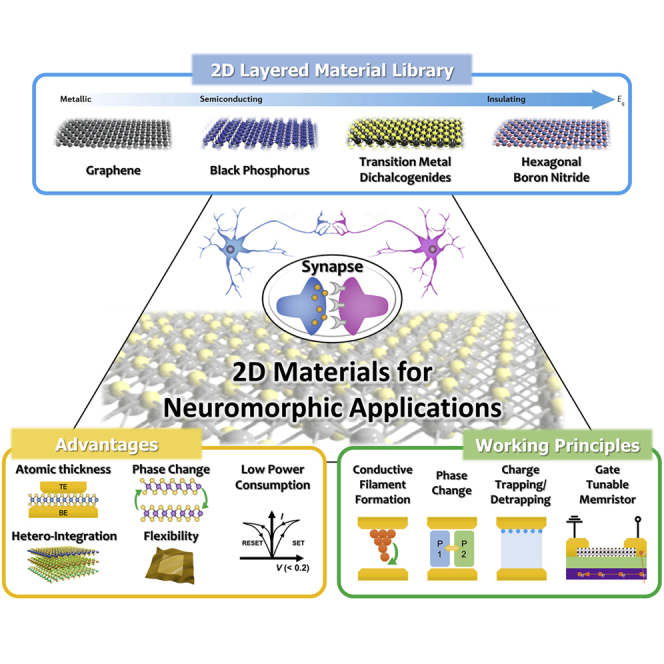

## Introduction

The past couple of decades have been characterized by drastic technological advances leading to the emergence of the era of “big data,” necessitating the study and development of technologies capable of handling colossal amounts of data (LeCun et al., 2015). There have been extensive studies and significant breakthroughs in the field of artificial intelligence (AI) technology, which enables complex computation and processing of big data at human-level complexity (Lawrence et al., 1997; LeCun et al., 2015). These AI programs employ artificial neural networks (ANNs), which have been proved to outperform traditional algorithms; however, their computations are based on classic von Neumann architectures (Nawrocki et al., 2016). Since its inception, von Neumann architectures have been widely used in computing, yet they are characterized by physical separation and linear interaction between the logic and memory components with a slow data transfer rate leading to high power consumption, and thus affecting the overall efficiency (Von Neumann, 1993). Such inefficiency and high power consumption (e.g., up to 1 MW for Google AlphaGo, Silver et al., 2016) associated with the von Neumann architecture have led to the research into neural network architectures inspired by the human brain with lower power consumption and the ability to perform complex tasks across a wide range of sectors including robotics, health care, security, internet of things, transportation, and manifold beyond (Sangwan and Hersam, 2020; Upadhyay et al., 2016).

The human brain is a complicated neural network consisting of ∼10^11^ neurons connected by ∼10^15^ synapses with the ability to process a greater quantity of information with a considerably lower power consumption of ∼20 W (Hart, 1983; Tian et al., 2015). The human brain outperforms conventional computers for complex tasks owing to its distinct architecture and functionality: (1) it is profoundly compact, with parallel computation and three-dimensional organization; (2) it hosts co-location of logic and memory; (3) it has high power efficiency; and (4) it is self-learning with the ability to adapt to environmental changes (Kandel et al., 2000; Kuzum et al., 2013). In neuro-inspired architectures, the process of learning and extraction is carried out by the synaptic weight updates. It is observed that the arrays implemented with synapses with hundreds of conductance states will show improved learning capability, which leads to enhancement in the robustness of the network (Yu, 2018). Another key feature of interest is the dynamic range, which is defined as the ON/OFF between the minimum and maximum conductance of the synapse. A broad dynamic range (>100) is imperative, which allows superior mapping capability of the synaptic weights to the conductance states in an algorithm (Yu, 2018). Furthermore, the trends observed in the synaptic weight change define the features of asymmetry and linearity in the conductance change. Such conductance variation, which stems from the response to the input programming pulses, plays a critical role in the training of the neural network.

The early successes in such neuromorphic computer architectures have been achieved in the hardware neural networks (HW-NNs) to realize brain-like computing based on non-von Neumann architectures. The brain carries out its complex computations and information flow via the two basic computational units in the brain, namely, the neurons and the synapses (Kuzum et al., 2013). These computations involve integrating inputs coming from other neurons, which leads to the generation of spikes and changes in the strength of synapses (synaptic weight) as a result of the neuronal activity (Abbott and Regehr, 2004). Similarly, HW-NNs are composed of multiple artificial synapses that emulate the behavior of the biological synapses in the human brain by constantly memorizing and updating the internal conductivity (“synaptic weight”) in response to changes in the system arising from external stimuli (Kim et al., 2017; Pastrana, 2010). The IBM TrueNorth developed in 2014 is a prominent example among HW-NNs (Merolla et al., 2014), which was successfully demonstrated for image recognition. However, this was based on conventional electronic materials, specifically, conventional complementary metal-oxide-semiconductor (CMOS) neural networks. Such HW-NN founded on CMOS technology carries out synaptic operation based on volatile random access memory (RAM), which is highly energy consuming and negates the intrinsic power consumption advantage associated with such brain-like systems.

Recent research efforts have been geared toward exploring different mechanisms and materials for the implementation of non-volatile memory (NVM)-based HW-NNs with improved capabilities, high energy efficiency, and scalability. Such endeavors have led to the emergence of different technologies and materials that can produce both memory and storage units, such as resistive RAM (ReRAM) (Jo et al., 2010; Prezioso et al., 2015; Strukov et al., 2008; Yu et al., 2011), phase change memory (Burr et al., 2015; Tuma et al., 2016; Wong et al., 2010), spin-torque transfer memory (Sengupta and Roy, 2018), and conductive bridging memory (Kang et al., 2019; Suri et al., 2013). Memristors have been considered as promising candidates among an array of NVM technologies owing to their markedly smaller footprint and high energy efficiency. Conventional implementation of these memristors employs bulk materials (e.g., transition metal oxides). However, patterning these materials into small footprints ultimately limits the control over their neuromorphic functionality because the device performance of memristors is strongly affected by the atomic-scale defects present in them. Furthermore, the conventional materials employed in previously explored memristors are intrinsically limited due to their bulky volume as well as structural and chemical variations at the nanoscale, leading to unreliable switching performance. Hence, considerable research efforts have been dedicated toward the implementations of memristors using low-dimensional materials with high crystallinity endowing favorable hallmarks of monolayer limit, quantum phase transitions, controllable defects, as well as stoichiometry, which in turn enable precisely controlled synaptic behavior (Jariwala et al., 2014; Sangwan and Hersam, 2018).

Recently, two-dimensional (2D) materials including graphene (Geim and Novoselov, 2007), transition metal dichalcogenides (TMDs) (Bhimanapati et al., 2015; Chhowalla et al., 2013; Jariwala et al., 2014; Ko et al., 2020; Wang et al., 2020), hexagonal boron nitride (h-BN) (Song et al., 2010; Zheng et al., 2018), black phosphorous (BP) (Xia et al., 2014), and MXenes (Anasori et al., 2017; Lei et al., 2015) have attracted substantial research attention among other nanomaterials due to their unique intrinsic properties. Their desirable properties, including atomic thickness, dangling-bond-free surfaces, mechanical strength, high integration density, tunable electrical transport, and optical properties, as well as low energy consumption, make them ideal candidates for applications in a wide range of electronic devices (Gupta et al., 2015; Mas-Ballesté et al., 2011; Xia et al., 2017). More recently, the applications of 2D materials have been extensively studied for energy-efficient and high-performing artificial synapses (Arnold et al., 2017; Chen et al., 2019c; Dev et al., 2020; Hu et al., 2019; Jiang et al., 2017; Kalita et al., 2019; Kim et al., 2019c; Krishnaprasad et al., 2019; Kumar et al., 2019; Li et al., 2018; Liu et al., 2019; Mao et al., 2019; Paul et al., 2019; Pradhan et al., 2020; Xie et al., 2018a, 2018b; Xu et al., 2019; Yan et al., 2019a; Yi et al., 2018; Zhu et al., 2019). Furthermore, owing to their dangling-bonds-free surface and atomically thin nature, a variety of 2D materials-based heterostructures have been developed in spite of their lattice mismatch (Novoselov et al., 2016). 2D layered materials can be utilized as active materials in diode-type neuromorphic device architecture with two-electrode configuration, allowing for the storage and updating of synaptic weights via various mechanisms, e.g., the formation of filaments (Lee et al., 2018; Shi et al., 2018; Xu et al., 2019), the transformation of phases (Zhu et al., 2019), or redistribution of atomic vacancies (Zhu et al., 2019). The unrivaled thinness of 2D materials also enables rapid electrical switching in memristor devices circumventing short channel effects, leading to improved energy efficiency (Stanford et al., 2018). Additionally, 2D materials and their heterostructures can be integrated into transistor-type devices to achieve desired synaptic characteristics owing to the absence of surface/interface defects allowing for precise modulation of surface/interfacial traps (Liu et al., 2019; Seo et al., 2018). Therefore, 2D materials hold tremendous prospects for neuromorphic applications in terms of scalability, learning, and energy efficiency.

**Figure 1:**
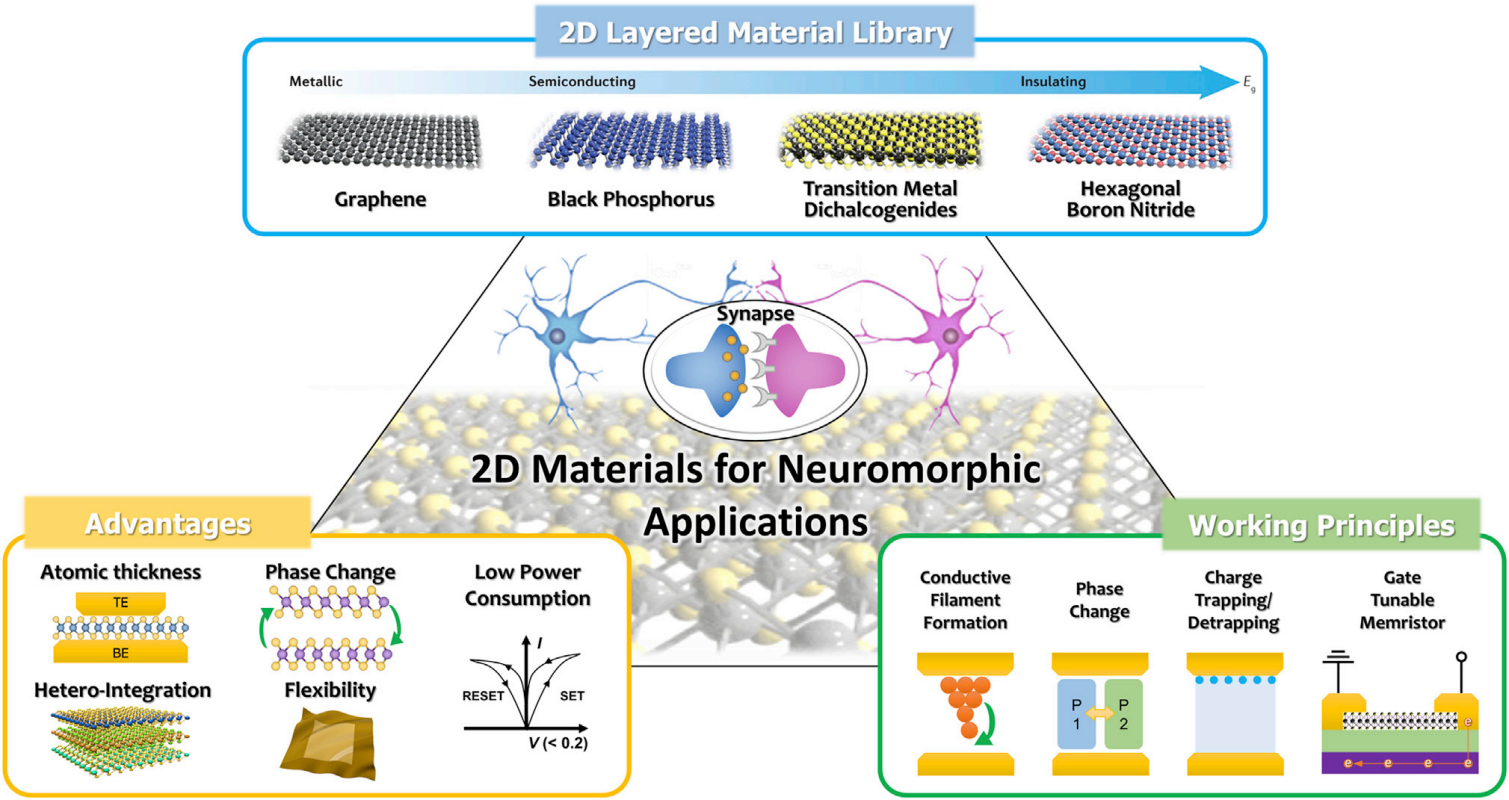
Overview of 2D Materials for Neuromorphic Applications Models of the 2D layered material library are reprinted with permission from (Liu et al., 2016). Copyright 2016 Springer Nature.

In this review, the unique properties of 2D materials for high-performance neuromorphic computing are primarily demonstrated, as overviewed in Figure 1. Various operating principles of 2D materials-enabled neuromorphic devices and their performance demonstrations are comprehensively presented. Lastly, the current challenges facing the successful integration of 2D materials in neuromorphic devices and future prospects for their implementation for brain-like computation are discussed.

## Promise of 2D Materials for Neuromorphic Application

To date, a variety of 2D materials adopting planar structure have been explored for neuromorphic applications benefiting from their atomic-scale small thickness, dangling-bonds-free surfaces, and superiority in upscale device fabrications. The 2D layered nanomaterial family exhibits a great diversity of electrical properties, encompassing conductors (e.g., graphene), semiconductors (e.g., TMDs and BP), and insulators (e.g., h-BN). Thus, broad applicability has been suggested for various components in neuromorphic devices. For example, semi-metallic graphene with zero band gap and extremely high carrier mobility (Geim and Novoselov, 2007) was reported to be suitable for electrodes of memristive devices presenting ultralow switching power and nonlinear I-V characteristics due to its low density of states, which inhibits efficient carrier injection (Qian et al., 2014). As another example, h-BN was applied as an active layer for memristive memory devices, exhibiting reproducible switching endurance as well as long retention time (Qian et al., 2016). Recent decades have also witnessed TMDs being widely explored for switching devices based on their diverse electrical property breadths. In general, TMDs are mainly composed of transition metals (group IV to group VIII, e.g., molybdenum [Mo], tungsten [W], platinum [Pt]) and chalcogens (e.g., sulfur [S], selenium [Se], tellurium [Te]), with approximately over 80 types experimentally developed so far. They exhibit a wide spectrum of conducting behaviors due to diverse elemental combinations, ranging from semi-metallic (e.g., palladium ditelluride [PdTe_2_], platinum ditelluride [PtTe_2_]) to semiconducting (e.g., molybdenum disulfide [MoS_2_], tungsten diselenide [WSe_2_]) (Ko et al., 2020). Initial efforts were devoted to exploring semiconducting 2D TMD layers (e.g., MoS_2_), which has then evolved to insulating materials (e.g., h-BN) as well. The main advantages of 2D TMDs as neuromorphic channels over conventional materials (e.g., metal oxides) include (1) layered “crystallinity” for reliable operation versus “amorphous” metal oxides that are difficult to spatially control the pathways of charge carriers due to stochastic nature and (2) extremely small thickness that is advantageous for faster switching, higher density, and lower energy consumption. Although such structural advantages are commonly shared in all 2D TMDs, it is noted that the specific charge transport mechanisms responsible for neuromorphic applications may vary with their constituent elements, determined by how the devices are operated. In fact, various working principles and structure-property relations exist despite the structural similarity of 2D TMDs. For instance, 2D TMD-based vertical memristive devices are able to tune multiple resistances by forming and rupturing conductive filaments through the migration of ions stemming from their electrodes (Dev et al., 2020; Xu et al., 2019). The spatial pathways of these ions can be further tuned by the intrinsically existing in-plane grain boundaries of 2D TMD layers (Sangwan et al., 2015), as well as by the externally introduced intercalations in between each 2D layer (Zhang et al., 2019; Zhu et al., 2019). A comprehensive review of various TMD-based switching mechanisms is found in the following section.

**Figure 2:**
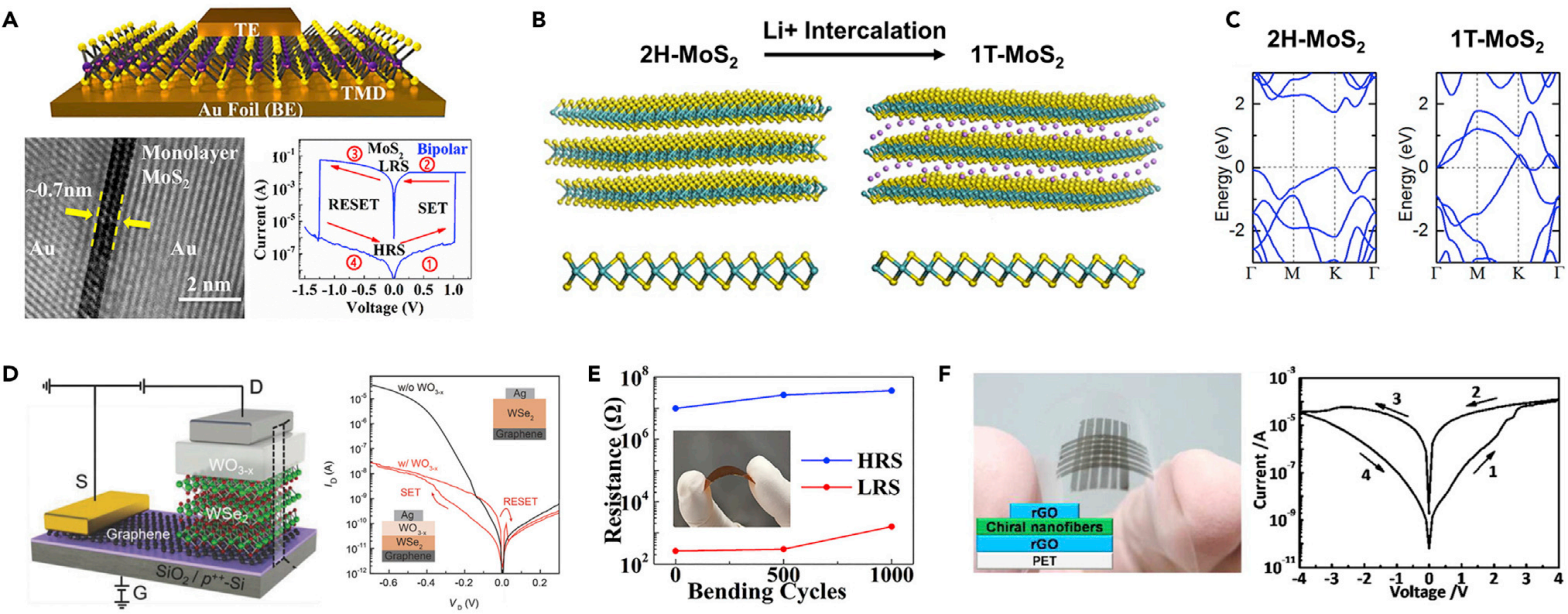
Promise of 2D Materials for Neuromorphic Applications (A) (Top) Schematic illustration of TMD lithography-free and transfer-free sandwich (top and bottom gold electrodes) based on MoS_2_ grown on Au foil. (Bottom-left) A cross-sectional TEM image of Au/MoS_2_/Au litho-free device revealing the atomically sharp and clean monolayer interface. (Bottom-right) Representative I-V curve of bipolar resistive switching behavior in a monolayer MoS_2_ crossbar device. Reprinted with permission from (Ge et al., 2018). Copyright 2018 American Chemical Society. (B) Phase change behavior of trigonal prismatic 2H-MoS_2_ to octahedral 1T-MoS_2_ through Li^+^ intercalation. Reproduced with permission from (Fan et al., 2015). Copyright 2015 American Chemical Society. (C) Electronic band structure of 2H-MoS_2_ (semiconducting) and 1T-MoS_2_ (metallic). Reprinted with permission from (Gao et al., 2015). Copyright 2015 American Chemical Society. (D) (Left) Schematic representation of synaptic barristor consisting of vertically integrated WO_3–x_ memristor and WSe_2_/graphene barristor. (Right) I_D_-V_D_ curves showing the gate-tunable resistive switching characteristics of the devices with (red line) and without the WO_3–x_ layer (black line). Reprinted with permission from (Huh et al., 2018). Copyright 2018 WILEY-VCH Verlag GmbH & Co. KGaA, Weinheim. (E) Stable resistance of atomristor device on PET after 1,000 bending cycles at 1% strain with the high-resistance and low-resistance states. Reprinted with permission from (Ge et al., 2018). Copyright 2018 American Chemical Society. (F) (Left) Snapshot and schematic illustration of 6 × 6 flexible memory devices based on chiral MoS_2_ nanofibers on PET. (Right) Initial I-V characteristics of a chiral MoS_2_ nanofiber-based memory cell. Reprinted with permission from (Tan et al., 2015). Copyright 2015 American Chemical Society.

The extremely small thickness (less than several nm) of 2D material families provides distinct benefits for amplified switching performance. In particular, it ensures a shortened pathway for electric field-driven diffusion of charge carriers, which makes them particularly suitable for fast electrical switching and energy-efficient operation. Previous studies reported that the energy consumption for synaptic operation in 2D materials-adopted devices could be downscaled to femto Joule (fJ)-level per spike, thus being especially advantageous for neuromorphic applications (Paul et al., 2019; Seo et al., 2018; Zhu et al., 2018). In addition, the near-atom thickness of 2D materials also provides favorable opportunities for miniaturizing device units. Ge et al. were the first to introduce the memristor effect in an atomic sheet, which is called the atomristor, utilizing non-volatile resistance switching phenomenon in the atomic sheet of monolayer TMDs (i.e., MoS_2_, WSe_2_, molybdenum diselenide [MoSe_2_], and tungsten disulfide [WS_2_]), as shown in Figure 2A (Ge et al., 2018). Moreover, 2D materials exhibit defect-mediated surfaces (e.g., saturated dangling bonds at the basal plane) with minimal structural/chemical changes compared with conventional metal oxides, therefore enabling the high controllability of spatially defined conductive paths. Their planar structure with a high surface-to-volume ratio also allows for unique defect engineering schemes. Defect generation enables their applications for cation-based resistive switching devices, which would otherwise be unfeasible due to their dense planar structure impermeable for ions/molecules (Zhao et al., 2018).

Characteristic phase change behaviors in 2D materials, especially in 2D TMDs, were also subject to extensive investigations for potential suitability for neuromorphic applications. Previous studies on the phase engineering of 2D materials revealed that the electrical properties of 2D TMD materials could be tuned using various techniques, uncovering unique phase change behaviors. For example, MoS_2_, which is among the best-known representative 2D TMD materials, transforms its crystal structure from the original trigonal prism (2H) into an octahedron (1T) through the intercalation of ions, resulting in the semiconducting-to-metallic transition (Figures 2B and 2C) (Acerce et al., 2015; Fan et al., 2015; Gao et al., 2015; Kan et al., 2014). Furthermore, other studies have also demonstrated that various methods, including charge transfer, irradiations, and stress inductions, can also induce phase change behavior in 2D TMD materials, which provide extended opportunities for the development of phase-change-based memristor devices (Cho et al., 2015; Xiao et al., 2019; Zhu et al., 2019).

The electrical properties of 2D materials can be further diversified through their heterolayer integration enabled by van der Waals (vdW) bonding, avoiding the undesirable epitaxial constraints demanded in conventional materials (Geim and Grigorieva, 2013; Novoselov et al., 2016). Additionally, as memristive devices based on 2D materials are mostly fabricated with a vertical architecture consisting of the two electrodes and the sandwiched active layer between them, 2D heterolayer-based synaptic architecture (diode-type or transistor-type) can be scaled down to sub-10 nm of thickness, which has the advantage of a low switching voltage (Figure 2D) (Huh et al., 2018).

Furthermore, the 2D materials-based memristive devices exhibit opportunities for flexible and wearable neuromorphic technologies such as neuro-prosthetics due to the inherently thin architecture (sub-10 nm level of thickness) and mechanical resilience of 2D materials (Ko et al., 2020). The past decade has witnessed a growing range of research endeavors on the development of 2D materials-based flexible memristive devices with polymeric substrates (e.g., polyethylene terephthalate [PET], and polyimide [PI]) (Liu et al., 2012; Tan et al., 2015). For example, an atomristor device based on MoS_2_ and Au electrodes integrated on a flexible PET substrate retained its memristive behavior even after a large number of mechanical bending (Figure 2E) (Ge et al., 2018). An additional demonstration of flexible devices based on reduced graphene oxide (rGO) and MoS_2_ on PET substrate also exhibited the memristive effect (Figure 2F) (Tan et al., 2015).

Overall, owing to the low thickness and unique planar structure, 2D materials-based memristive devices possess multiple desirable advantages for neuromorphic computation. The resistive switching mechanisms of 2D materials, including vdW heterostructures, are introduced in the following section.

## Working Principle of 2D Material-Based Neuromorphic Devices

### Phase Change Memristors

**Figure 3:**
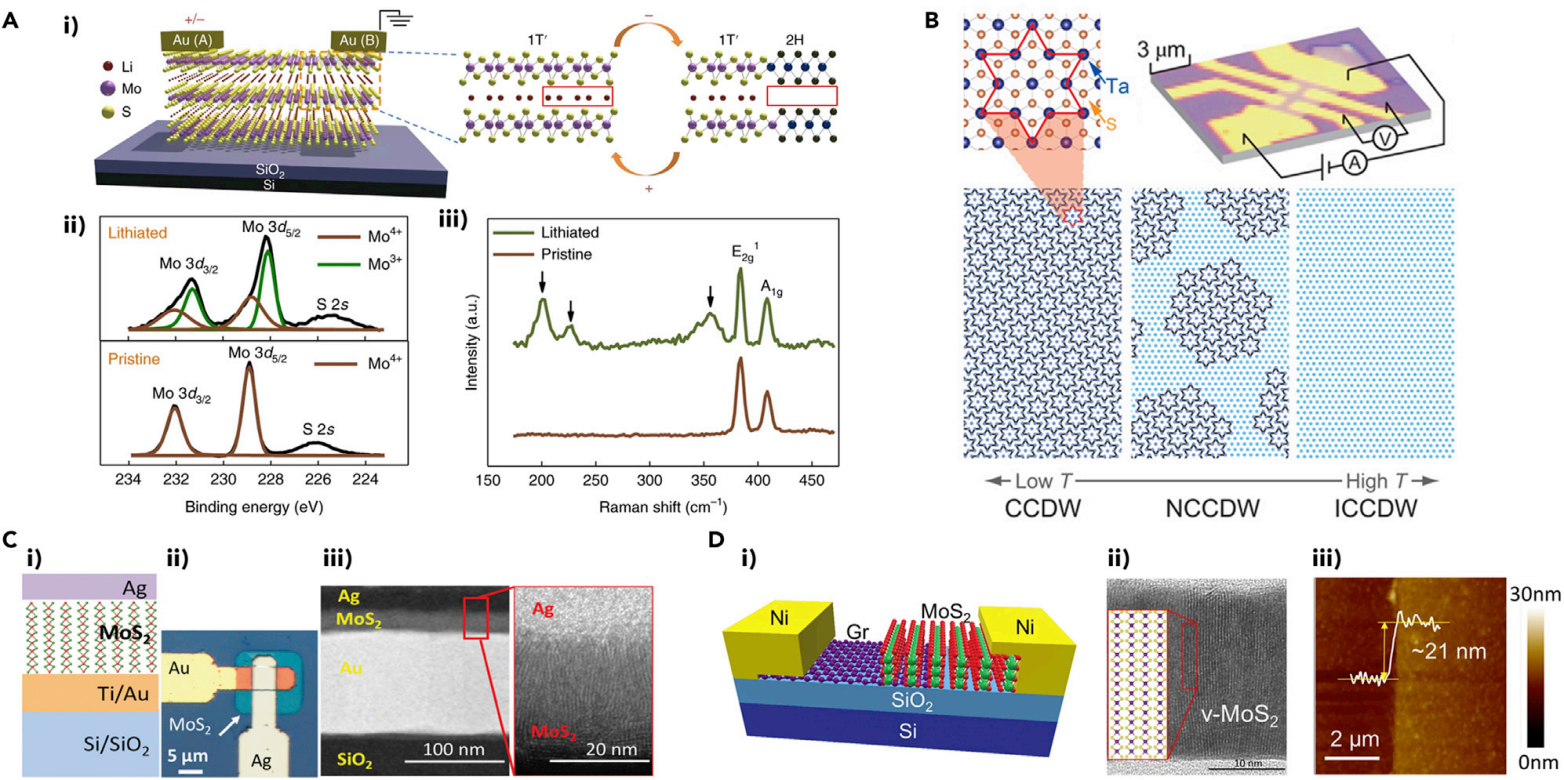
Representative Images Showing the Working Principles of Synaptic Devices: Phase Change Memristors, Quantum Phase Transition Memristors, and Resistive Memory Devices (A) (i) Schematic illustration of memristive behavior in MoS_2_ using electric field-controlled reversible 2H/(1T′) to 1T′/(2H) phase transition controlled by Li^+^ concentration. (ii) XPS spectra for the lithiated and pristine films. The lithiated film displaying the appearance of Mo^3+^ peaks indicating conversion of Mo^4+^ to Mo^3+^ peaks. (iii) Raman spectra of lithiated and pristine MoS_2_ films showing additional peaks (marked by arrows) at 200, 225, and 355 cm^−1^, corresponding to the characteristic peaks of 1T′ MoS_2_. Reprinted with permission from (Zhu et al., 2019). Copyright 2019 Springer Nature. (B) An optical microscopic image of a 1T-TaS_2_ memristive device (top-right) and a schematic illustration of Ta atom network in the CCDW (left), hexagonal NCCDW (middle), and ICCDW (right) phases, with the CCDW phase zoomed-in in the inset. The dark blue dots represent the displaced Ta atoms from their undistorted lattice coordinates, forming David-star clusters. Reproduced with permission from (Yoshida et al., 2015). © The Authors, some rights reserved; exclusive licensee American Association for the Advancement of Science. Distributed under a Creative Commons Attribution NonCommercial License 4.0 (CC BY-NC) http://creativecommons.org/licenses/by-nc/4.0/. (C) (i) Schematic illustration and (ii) optical microscopic image of the vertical stack MoS_2_ threshold switching memristor. (iii) Cross-sectional STEM and HRTEM images of the MoS_2_ memristive device showing vertically aligned 2D MoS_2_ layers. Reprinted with permission from (Dev et al., 2020). Copyright 2020 IEEE. (D) (i) Schematic illustration of vertical MoS_2_/graphene threshold switching memristor. (ii) Cross-sectional HRTEM image of vertically aligned MoS_2_ at the MoS_2_/graphene interface. (iii) AFM image revealing MoS_2_ thickness of ∼21 nm. Reprinted with permission from (Kalita et al., 2019). Copyright 2019 Springer Nature.

A large number of 2D TMDs possess intrinsic advantages of structurally and/or electrically controllable phase change characteristics because they exist in various polymorphs. For memristive applications, it is critically important to precisely control the duration of phase change as well as its process reversibility. Zhu et al. demonstrated a controlled reversible phase transition of MoS_2_ in a localized area by driving lithium ions (Li^+^) with an external electric field (Zhu et al., 2019). Figure 3A(i) shows the schematic illustration describing the phase transition, in which Li^+^ migration is driven by the electrode labeled as A. An increase in Li^+^ concentration leads to the 1T′ phase and a decrease in Li^+^ concentration brings forth the 2H phase. A possible explanation for the 2H to 1T′ transition is that the intercalated Li atoms donate electrons to the Mo 4d orbitals, making the 2H phase unstable. This claim was corroborated by X-ray photoelectron spectroscopy (XPS), as depicted in Figure 3A(ii), which reveals that lithiated MoS_2_ possesses a portion of the Mo^4+^ converted to Mo^3+^. Additionally, Raman spectra in Figure 3A(iii) show additional peaks at 200, 225, and 355 cm^−1^ emerging after lithiation, which correspond to the MoS_2_ 1T′ peaks. This lithiation process in MoS_2_ is possible due to the high in-plane diffusivity of Li^+^ in MoS_2_ (Stephenson et al., 2014). Zhang et al. deployed the phase transition of molybdenum ditelluride (MoTe_2_), another example of polymorphic TMD (Cho et al., 2015), to devise phase change resistive memories. After the application of the electric field, the semiconducting 2H phase of MoTe_2_ transitions into a distorted 2H phase, which exhibits electronic characteristics that lie between the semiconducting 2H phase and the metallic 1T′ phase (Zhang et al., 2019).

### Quantum Phase Transition Memristors

1T-tantalum disulfide (TaS_2_) is a layered 2D material system with first-order charge density wave (CDW) phase transitions (Sipos et al., 2008; Stojchevska et al., 2014). Yoshida et al. demonstrated multi-states memristive non-volatile switching in 1T-TaS_2_, which showed the first-order CDW phase transitions (Figure 3B). Few-layer 1T-TaS_2_ synthesized by a vapor transport method exhibited the switching from incommensurate CDW (ICCDW) to a nearly commensurate CDW (NCCDW) phase at around 350 K, and another phase transition from an NCCDW to the Mott state commensurate CDW (CCDW) phase from 100 to 220 K, with respect to temperature sweep direction. These transitions led to hysteretic current-temperature curves and memristive current-voltage characteristics. Figure 3B shows that 13 Ta atoms form a David-star cluster in the CCDW phase as zoomed-in in the inset. The neighboring NCCDW phase has a hexagonal arrangement that originated from CCDW domains, which transforms into the ICCDW phase upon further heating (Yoshida et al., 2015).

### Resistive Memory Device

Memristive devices are generally in a two-terminal configuration, similar to resistive switching devices. Even though three-dimensional (3D) materials have been extensively adopted for them, it is beneficial to investigate 2D materials for their continued miniaturization to the true nanoscale. Depending on the switching characteristics of the memristive devices, fundamental devices of neuromorphic architecture, i.e., synapses and neurons, can be emulated. For instance, non-volatile memristors were utilized to implement synapses, whereas volatile memristors were used to demonstrate neurons. 2D materials in memristive devices can be of either vertical or lateral orientation and may also be a mixture of both. Vertical memristive devices employing 2D materials can be scaled down to sub-10 nm thickness achieving high integration density and small switching voltage, and hence can be useful in low-power applications. Electrode materials play a crucial role in the resistive switching of memristors. Dev et al. demonstrated a threshold switching memristor (TSM) for artificial neuron, using vertically aligned MoS_2_ layers grown by a chemical vapor deposition (CVD) method (Dev et al., 2020). The schematic and optical imaging of the TSM is provided in Figure 3C(i) and (ii), respectively. The switching mechanism is ascribed to the facile diffusion of Ag conductive filaments within the electrochemically active vertically aligned MoS_2_ layers driven by the electric field. Upon removal of the electric field, the Ag conductive filament turns back to a spherical shape to reduce interfacial energy, resulting in the non-volatile characteristics of the device. The orientation of the grown MoS_2_ films was confirmed by transmission electron microscopic (TEM) analysis, including dark-field scanning TEM (STEM) and bright-field high-resolution TEM (HRTEM) showing well-resolved vertically aligned 2D layers as depicted in Figure 3C(iii). Kalita et al. demonstrated artificial neuron using vertical MoS_2_/graphene TSM, as illustrated in Figure 3D(i) (Kalita et al., 2019). In this approach, CVD-grown large-area graphene was wet-transferred onto a silicon dioxide/silicon (SiO_2_/Si) wafer followed by CVD sulfurization of pre-patterned Mo, resulting in a heterostructure of MoS_2_/graphene. Nickel electrodes were subsequently deposited and patterned as top contacts. The cross-sectional HRTEM image in Figure 3D(ii) shows vertically aligned 2D MoS_2_ layers on top of graphene with a high density of exposed edge sites. Furthermore, the thickness of the MoS_2_ film was ∼21 nm as characterized by atomic force microscopy (AFM) (Figure 3D(iii)), which is in agreement with the cross-sectional TEM observation. It was suggested that the probable switching mechanism is the facilitated migration of oxygen ions along the vertical grains of MoS_2_ with a large density of grain boundaries. Pan et al. realized the resistive switching effect in CVD-grown multilayer insulating h-BN. Studying with a different electrode material, they concluded that the switching behavior was due to the migration of metallic ions from electrodes along the grain boundary and boron vacancies in CVD-grown h-BN, similar to the behavior observed in CVD-grown MoS_2_ (Pan et al., 2017). As forementioned, bendability is an intrinsic advantage of 2D materials, offering opportunities for mechanically flexible resistive switching devices. Siddiqui et al. developed h-BN-polyvinyl alcohol composites-based resistive switching devices via a solution-based method, where the h-BN flakes were extracted by liquid exfoliation. The devices showed excellent bendability, maintaining initial characteristics up to 1,500 bending cycles (Siddiqui et al., 2017). Liu et al. demonstrated MoS_2_-polyvinylpyrrolidone (PVP)-based resistive switching devices, where the MoS_2_-PVP composites were prepared using a solution-based process (Liu et al., 2012).

### Atom Switches

**Figure 4:**
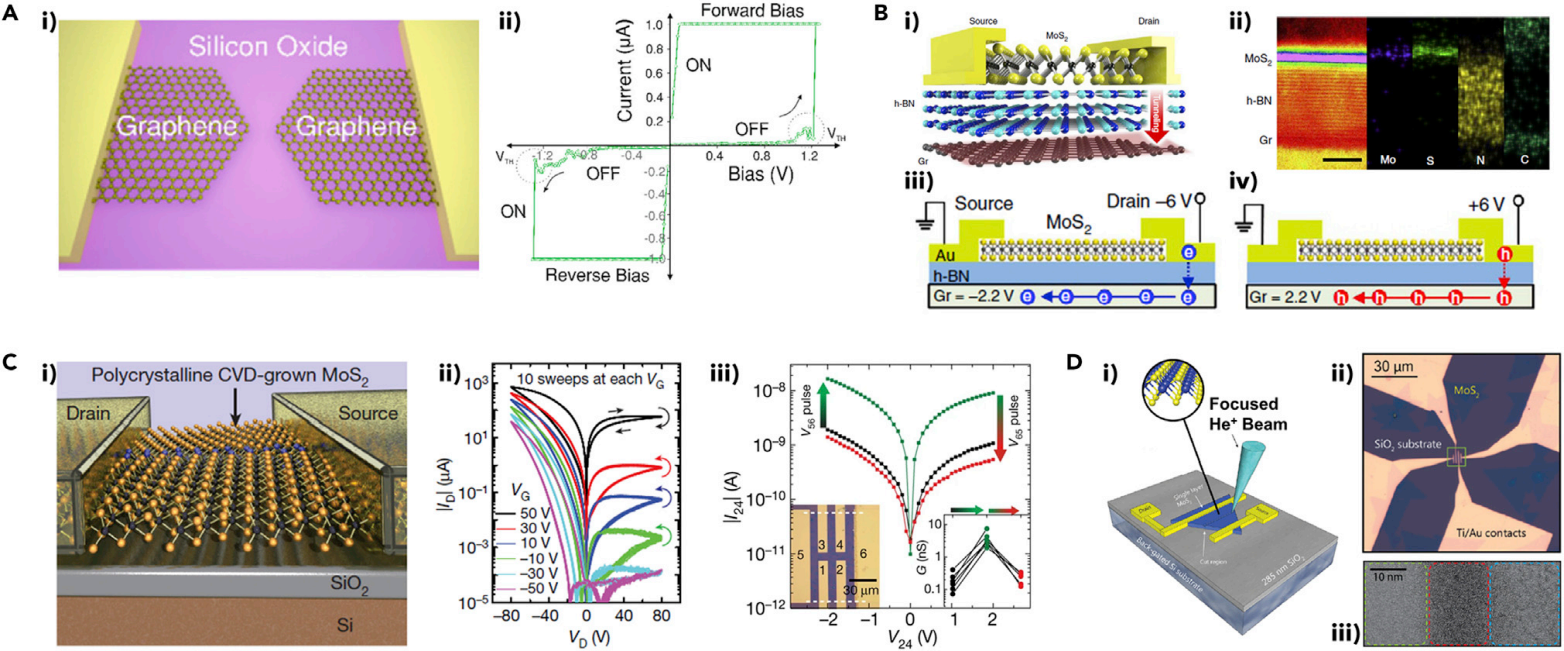
Representative Images Showing the Working Principles of Synaptic Devices: Atom Switches, Charge Trapping/Detrapping, Gate-Tunable Memristors, Defect (Vacancies) Engineering (A) (i) Schematic representation of a graphene-based atom switch using a lateral nanogap. (ii) I-V characteristics of a graphene nanogap (3 nm) atom switch during the low-bias switching in ambient conditions. Reprinted with permission from (Sarwat et al., 2017). Copyright 2017 American Chemical Society. (B) (i) Schematic representation of a diode-type 2D vertically stacked MoS_2_/h-BN/graphene heterostructure (two-terminal floating-gate memory) via charge trapping/detrapping. (ii) Cross-sectional bright-field STEM and EDS elemental mapping of the device with 10-nm hr-BN. (iii) Schematic illustration of electron tunneling between the electrode and the graphene floating gate that shows the memory operation during a program state. (iv) Schematic illustration of the memory operation during an erase state. Reprinted with permission from (Vu et al., 2016). Copyright 2016 Springer Nature. (C) (i) Schematic illustration of a CVD-grown 2D monolayer MoS_2_ memtransistor device built on SiO_2_ (300 nm) on doped Si (gate). (ii) I_D_-V_D_ curves for 10 consecutive sweeps at each gate bias (V_G_) that show the gate-tunability. (iii) I-V curve between terminals 2 and 4 of a six-terminal MoS_2_ memtransistor (left inset) at constant V_G_ = 20 V. The right inset showing heterosynaptic plasticity observed by reversibly changing the conductance. Reprinted with permission from (Sangwan et al., 2018). Copyright 2018 Springer Nature. (D) (i) Schematic representation of the irradiation strategy for post-growth defect engineering. (ii) Optical micrograph of a CVD-grown 2D monolayer MoS_2_ memtransistor fabricated by localized He^+^ beam irradiation. (iii) TEM image of the irradiated (red) fissure region and adjacent (green and blue) regions on a mechanically exfoliated suspended few-layer MoS_2_ sample. Reprinted with permission from (Jadwiszczak et al., 2019). Copyright 2019 American Chemical Society.

Atom switches are another example of novel resistive switching devices where 2D nanomaterials-based applications receive ongoing research attentions. Graphene-based atom switches are based on the formation of a carbon atomic chain in a nanojunction or a nanogap by an applied electric field in graphene (Sangwan and Hersam, 2020; Sarwat et al., 2017; Standley et al., 2008). Graphene-based nanogaps offer means to reliably contact nanoscale objects that are difficult to attain with conventional 3D metal electrodes (Sarwat et al., 2017). Stanley et al. reported graphene-based atomic-scale switches (non-volatile memory) employing graphene break junctions (Standley et al., 2008). They demonstrated the operation of graphene switches by creating nanoscale gaps using electrical breakdown (Khondaker and Yao, 2002; Park et al., 1999) on graphene sheets. By applying appropriate bias voltage pulses, the conductance between the gap switched between high (ON) and low (OFF) conductance states (Standley et al., 2008). Sarwat et al. reported consistent voltage-driven switching in sub-5 nm graphene nanogaps and reversible resistance switching in ambient conditions (Sarwat et al., 2017). Figure 4A(i) shows a schematic representation of a graphene nanogap atom switch. Nanogaps (∼1–60 nm) were created via a feedback-controlled electro-burning method. As shown in Figure 4A(ii), the device switched from a high resistive state to a low resistive state in ambient conditions at a switching voltage (forward bias) of 1.22 V and a current of 60 nA (the first quadrant). The third quadrant in Figure 4A(ii) shows the same device under reverse (negative) polarity switching at a voltage of 1.28 V and a current of 100 nA. In this device, the resistance switching is believed to be entirely dominated by the formation of nanoscale carbon filaments (Sarwat et al., 2017).

### Charge Trapping/Detrapping

Charge trapping/detrapping mechanism has been reported in various 2D vdW structures, which employs trapping or detrapping electrons in a charge-trapping interfacial layer known as the weight control layer (WCL) (Seo et al., 2018). In transistor-type 2D vdW synaptic devices, the width of the tunneling barrier is increased or decreased by the trapped or detrapped electrons. Kumar et al. demonstrated robust two-terminal memristors based on zinc oxide (ZnO) and WS_2_ layers prepared by a radio frequency sputtering process (Kumar et al., 2019). The interlayer separation between ZnO and WS_2_ layers provided an effective porous medium for the growth of defective ZnO (Zn metal-rich and non-uniformly distributed oxygen vacancies), which provided a unique charge trapping/detrapping platform layer (Kumar et al., 2019). Vu et al. reported a two-terminal floating-gate memory based on a vertically stacked vdW heterostructure fabricated by a monolayer MoS_2_/h-BN/monolayer graphene (Vu et al., 2016), as shown in Figure 4B(i). The device is based on the tunneling-driven charge trapping/detrapping mechanism, where graphene is used as the WCL (floating gate). Figure 4B(ii) shows a cross-sectional bright-field STEM image and energy dispersive X-ray spectroscopy (EDS) elemental mapping revealing each layer of the device. In the reported heterostructure-based two-terminal tunneling RAM, the highly resistive 2D MoS_2_ monolayer allowed the storing of charges in graphene as it competed with the tunneling probability through the thin graphene where the appropriate thickness of the crystalline h-BN layer was necessary to invoke the asymmetric potential drop (Vu et al., 2016). At −6 V on drain bias, electrons are tunneled from drain to monolayer graphene (floating gate) and trapped in graphene due to a small potential drop at the contact, as shown in Figure 4B(iii). In contrast, at +6 V on drain bias, holes are trapped in graphene by tunneling from the drain, generating a highly conductive inversion channel in MoS_2_, as shown in Figure 4B(iv). The memory device demonstrated a large dynamic range (>10^9^), a long retention period (>10^4^ s), stable endurance (>10^5^ cycles), multilevel conductance, and an ultimately low off-state current of 10^−14^ A, leading to an ultrahigh ON/OFF ratio of over 10^9^ (Vu et al., 2016). Seo et al. demonstrated an optic-neural synaptic (ONS) device by implementing synaptic and optical-sensing functions together on the h-BN/WSe_2_ heterostructure (Seo et al., 2018). They created a charge-trapping layer on top of the h-BN for the adjustment of the WSe_2_ channel conductivity. The device is based on the trapping or detrapping of electrons in the WCL on h-BN with an O_2_ plasma treatment, which modulates the WSe_2_ channel conductivity (Seo et al., 2018). Many other studies also utilized 2D MoS_2_ layers in heterostructures based on charge trapping/detrapping mechanisms (Chen et al., 2019a; Kim et al., 2019c; Paul et al., 2019; Wang et al., 2019b; Yi et al., 2018).

### Gate-Tunable Memristors

Gate-tunable memristors, or memtransistors, are realized by combining the concepts of both transistor and memristor into a single device. Memtransistors offer both drain- and gate-tunable NVM functions, which efficiently emulate the long-term potentiation (LTP)/depression (LTD), spike-amplitude, and spike-timing-dependent plasticity (STDP) of biological synapses (Wang et al., 2019a). Laterally configured 2D materials are beneficial for devising 2D vdW heterostructures-based memtransistors by vertically assembling 2D layers in an open architecture. Many studies employed CVD-grown monolayer MoS_2_ in lateral memtransistors (Jadwiszczak et al., 2019; Sangwan et al., 2015, 2018; Wang et al., 2019a; Xie et al., 2017). Sangwan et al. demonstrated memtransistors (gate-tunable memristors) by availing the atomically thin nature of MoS_2_, which enables tuning of the SET voltage by a third gate terminal in a field-effect geometry based on grain boundaries in a monolayer MoS_2_ (Sangwan et al., 2015). The switching characteristics were found to be dependent on grain boundary topology, and the device utilized the rearrangement of atoms at certain grain boundaries in 2D monolayer MoS_2_ to reduce excessive leakage current in conventional oxide-based vertical metal-insulator-metal memtransistors (Sangwan et al., 2015). Additionally, Sangwan et al. reported multi-terminal memtransistors from a CVD-grown monolayer MoS_2_, as shown in Figure 4C(i). Figure 4C(ii) shows I_D_-V_D_ curves for 10 consecutive sweeps at each gate bias (V_G_) that show the gate-tunability. The six-terminal MoS_2_ memtransistors obtained via a scalable fabrication process (Figure 4C(iii)) showed gate-tunable hetero-synaptic functionality, which was not achievable in two-terminal memristors (Sangwan et al., 2018).

Wang et al. reported a multi-terminal memtransistor based on CVD-grown polycrystalline monolayer MoS_2_ by utilizing small grain sizes and ultrathin dielectrics (20 nm of hafnium dioxide [HfO_2_]) as the gate dielectric (Wang et al., 2019a). Moreover, post-growth defect engineering has shown promise for modifying (tailoring) the behavior of MoS_2_ memtransistors (Jadwiszczak et al., 2019; Xie et al., 2017), which will be discussed further in the next subsection. In addition to adopting 2D MoS_2_ layers, Yang et al. reported a three-terminal memtransistor employing mechanically exfoliated 2D gallium selenide (GaSe) nanosheets that served as the resistive switching layer (Yang et al., 2019b). The device showed non-volatile bipolar resistive switching characteristics, and the low migration energy of the intrinsic Ga vacancy in the p-type GaSe layer enabled an ultralow threshold electric field of ∼3.3 × 10^2^ V cm^−1^ with the ON/OFF ratio approaching as high as 5.3 × 10^5^ (Yang et al., 2019b).

### Defect Engineering

In many 2D neuromorphic devices, interfacial oxide traps (or vacancy defects) are responsible for initiating resistive switching at small bias due to the exceptionally high surface-to-volume ratio and ultra-thinness of 2D materials. Post-growth defect engineering approaches were adopted to modulate such structural variations in 2D TMDs-based synaptic field-effect transistors (FETs) and multilevel FET-based memories (Arnold et al., 2017; Chen et al., 2017b; He et al., 2016). Resistive switching behaviors in monolayer MoS_2_ (He et al., 2016; Sangwan et al., 2015), few-layer MoS_2_ (Arnold et al., 2017; Wang et al., 2018), and few-layer WSe_2_ (Chen et al., 2017b) were explained based on charge transfers through interfaces and grain boundaries. Sangwan et al. verified that bias-induced motions of defects in polycrystalline monolayer MoS_2_ are responsible for the resistive switching behaviors. 2D planar geometry of monolayer MoS_2_ is beneficial for the fabrication of large-area integrated circuits and post-growth defect engineering (Sangwan et al., 2018). It was verified that the Schottky barrier at the MoS_2_/metal interface could be tuned by the local redistribution of defects, especially grain boundaries, in monolayer MoS_2_ (Sangwan et al., 2018). Additionally, post-growth defect engineering schemes on monolayer MoS_2_ memtransistors were demonstrated using helium-ion (He^+^) beam irradiation (Jadwiszczak et al., 2019) or electron-beam irradiation (Xie et al., 2017). Jadwiszczak et al. reported 2D monolayer MoS_2_ memtransistors fabricated by site-specific irradiation using the focused probe of He^+^ beam irradiation that created a nanometer-scale defect-rich region, as shown in Figure 4D (Jadwiszczak et al., 2019). Figure 4D(i) shows the illustration of the He^+^ beam irradiation process, and Figure 4D(ii) shows an optical micrograph of the fabricated device with multiple channels. Figure 4D(iii) shows a TEM image of the irradiated (red) and adjacent (green and blue) regions on a mechanically exfoliated suspended few-layer MoS_2_ sample, evidencing the creation of localized defects by He^+^ beam (Jadwiszczak et al., 2019). Xie et al. also reported a 2D memristive transistor with short-term plasticity (STP) and demonstrated that local structural variations could reversibly modulate the drift of charges, enabling versatile memristive functionality (Xie et al., 2017). They incorporated 1T phase quantum dot superlattices on a 2H phase 2D monolayer MoS_2_ back-gated FET where the quantum dots functioned as charge-trapping sites upon irradiation of focused electron beam. The fabricated device demonstrated STP to light stimulation, gate-tunability, site controllability, light sensitivity, and room temperature operation. Table 1 summarizes the characteristics of resistive memory devices based on 2D materials.

**Table 1:** Memristive (Resistive Memory) Devices Based on 2D Materials

Working Principle	Active Layer	Electrode	Device	Growth/Fabrication Method	Power Consumption/Switching Voltage	Ref.
Phase change memristor	ML-MoS_2_	Au (top)	Phase change memristor	Mechanical exfoliation	6 V	(Zhu et al., 2019)
	2H (semiconductor) ↔ 1T′ (metal)					
Phase change memristor	ML-MoTe_2_	Ti/Ni (top), Ti/Au (bottom)	Resistive random access memory	Mechanical exfoliation	2.3 V	(Zhang et al., 2019)
	2H (semiconductor) ↔ 2H_d_ (transient state)					
Conductive filament	ML-MoS_2_	Ag/Au (top), Ti/Au (bottom)	Artificial neuron	CVD	0.35–0.4 V	(Dev et al., 2020)
Grain boundary-mediated transport	ML-MoS_2_/1L-graphene	Ni (top), graphene (bottom)	Artificial neuron	CVD/Wet transfer	2.8–2.9 V	(Kalita et al., 2019)
Conductive filament	BL-MoS_2_	Cu (top), Au (bottom)	Synapse	MOCVD/Layer-by-layer stack	0.1–0.2 V	(Xu et al., 2019)
Conductive filament	ML-h-BN	Ag (top), Cu (bottom)	Resistive memory	CVD/Wet transfer	0.72 V	(Qian et al., 2016)
Conductive filament/interface-mediated switching	ML-MoS_2_/1L-graphene	Ni/Au (top), graphene (bottom)	Synapse	CVD/Wet transfer	1.5 V	(Krishnaprasad et al., 2019)
Conductive filament	ML-h-BN-PVA composite	Ag (top), ITO (bottom)	Flexible resistive memory	Solution/liquid exfoliation	0.78 V	(Siddiqui et al., 2017)
Vacancy migration	ML-WS_2_	Pd (top), Pt (bottom)	Synapse	Solution	0.56–0.67 V	(Yan et al., 2019b)
Space-charge-limited current	ML-MoS_2_-PVA	Ag (top), Ag (bottom)	Flexible resistive memory	Solution/liquid exfoliation	3 V	(Rehman et al., 2016)
Space-charge-limited current	ML-MoS_2_-PVA	Al (top), rGO (bottom)	Flexible resistive memory	Solution/liquid exfoliation	3.5 V	(Liu et al., 2012)
Atom switch	1L- or BL-graphene	Au	Atomic-scale switches (lateral)	Electrical breakdown of graphene sheets/mechanical cleavage	–	(Standley et al., 2008)
Atom switch	ML-graphene	Au	Atom switches (lateral)	Feedback-controlled electro-burning/mechanical exfoliation	0.3 V	(Sarwat et al., 2017)
Charge trapping/detrapping	WS_2_/ZnO	Ag (top), Al (bottom)	Memristors	RF sputtering	0.8–1.6 V	(Kumar et al., 2019)
Charge trapping/detrapping	1L-MoS_2_/h-BN/1L-graphene	Cr/Au (top)	Two-terminal floating-gate memory	CVD/mechanical exfoliation	–	(Vu et al., 2016)
Charge trapping/detrapping	ML-WSe_2_/WCL/h-BN	Ti/Au (control), Pt/Au (synaptic)	Optic-neural synaptic device	O_2_ plasma treatment/residue-free transfer	66 fJ at 0.3 V pulse	(Seo et al., 2018)
Charge trapping/detrapping	ML-MoS_2_/h-BN/graphene/h-BN	Cr/Au (top), Si (bottom gate)	Human memory system programming: sensory memory, short-/long-term memory	Mechanical exfoliation/PVA transfer	64 pJ	(Chen et al., 2019a)
Charge trapping/detrapping	Au/h-BN/ML-MoS_2_/h-BN graphene or ML-MoS_2_/h-BN/ML-MoS_2_/h-BN	Cr/Au (top), p^++^-Si (bottom gate)	Multilevel optical memory	Mechanical exfoliation/PDMS stamping	–	(Kim et al., 2019c)
Charge trapping/detrapping	ML-MoS_2_/h-BN/graphene	Cr/Au (top), p^++^-Si (bottom gate)	Synaptic transistors	Mechanical exfoliation/dry transfer	5 fJ	(Paul et al., 2019)
Charge trapping/detrapping	ML-MoS_2_/PTCDA	Au (top), n^++^-Si (bottom)	MoS_2_/PTCDA heterojunction synapse	Mechanical exfoliation	10 pJ	(Wang et al., 2019b)
Charge trapping/detrapping	ML-MoS_2_/h-BN/h-BN/Au substrate	Au or Al (top), Au, Al, Pd, or highly doped Si (bottom gate)	Flash memory devices	Mechanical exfoliation	–	(Yi et al., 2018)
Gate-tunable memristor	1L-MoS_2_	Ti/Au (top S, D, gate)	Memtransistor	CVD	–	(Wang et al., 2019a)
Gate-tunable memristor	1L-MoS_2_	Au (top), Si (bottom gate)	Memtransistor	CVD	3.5–8.3 V	(Sangwan et al., 2015)
Gate-tunable memristor	ML-GaSe	Ag (side), Si (bottom gate)	Memtransistor	Mechanical exfoliation	0.8–1.8 V	(Yang et al., 2019b)
Gate-tunable memristor	ML-MoS_2_	Ti/Au (top), Si (bottom gate)	Memtransistor	Mechanical exfoliation	Dual gate: Light (0.63 mW)/∼3 V	(Yin et al., 2019b)
Gate-tunable memristor/defect engineering	1L-MoS_2_	Ti/Au (top), doped Si (bottom gate)	Memtransistor	CVD	Threshold voltage: 20 V (HRS), 10 V (LRS)	(Sangwan et al., 2018)
Gate-tunable memristor/defect engineering	1L-MoS_2_	Ti/Au (top), doped Si (bottom gate)	Memtransistor	CVD/focused He^+^ beam	16 nW (standby), 0.64 μW (operation)	(Jadwiszczak et al., 2019)
Gate-tunable memristor/defect engineering	1L-MoS_2_	Ti/Au (top), n^++^-Si (bottom gate)	Memtransistor	CVD/focused electron beam	–	(Xie et al., 2017)
Defect engineering	ML-MoS_2_	Ni (top), p^++^-Si (bottom gate)	Synaptic transistors	Mechanical exfoliation	–	(Arnold et al., 2017)
Defect engineering	1L-MoS_2_	- (top), n^++^-Si (bottom gate)	Non-volatile memory	CVD	5-9 V	(He et al., 2016)
Defect engineering	ML-MoS_2_	Graphene or Au (top and bottom)	Memristors	Mechanical exfoliation/PVA transfer	–	(Wang et al., 2018)
Defect engineering	ML-WSe_2_, ML-MoS_2_	Ti/Au (top), p^+^-Si (bottom gate)	Synaptic transistors (FETs)	Mechanical exfoliation	0.03–3 μW	(Chen et al., 2017b)

1L, one layer; ML, multi-layer; BL, bilayer; PTCDA, perylene-3,4,9,10-tetracarboxylic dianhydride; MOCVD, metal organic chemical vapor deposition; PDMS, polydimethylsiloxane; PVA, polyvinyl alcohol; HRS, high-resistance state; LRS, low-resistance state; RF, radio frequency

### vdW Heterostructure for Neuromorphic Applications

As forementioned, the unique planar structure, dangling-bond-free surfaces, and atomic thinness of 2D layered materials facilitate the integration of 2D heterostructures without lattice match and epitaxial constraints (Geim and Grigorieva, 2013; Novoselov et al., 2016). The assembly of various 2D layered materials into heterostructures has further diversified the synaptic applicability of 2D layered materials for neuromorphic computing (Chen et al., 2019a; Huh et al., 2018; Kalita et al., 2019; Paul et al., 2019; Seo et al., 2018; Tian et al., 2017a; Wang et al., 2018, 2019b). Various 2D layered materials have been employed as active channel, dielectric, and charge-trapping layers, leading to all-2D vdW heterostructure-based neuromorphic devices, owing to their advantages of ultra-thinness, excellent mechanical resilience, and optical transparency (Liu et al., 2012; Tan et al., 2015; Zhao et al., 2014).

**Figure 5:**
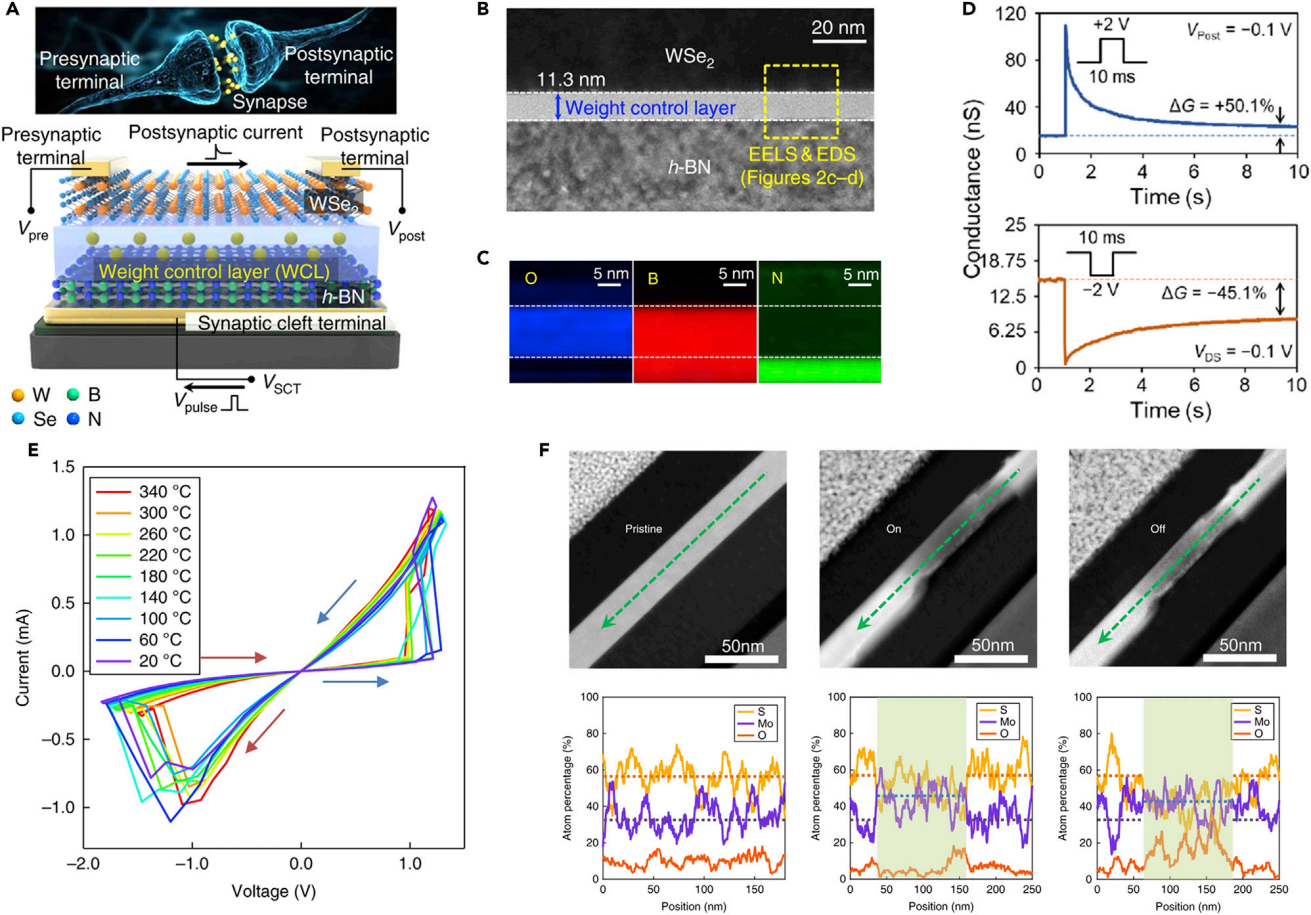
vdW heterostructures with 2D materials for neuromorphic applications (A) Schematic illustration of an h-BN/WCL/WSe_2_ synaptic device. (B and C) (B) Cross-sectional TEM image of the h-BN/WCL/WSe_2_ structure, and (C) electron energy loss spectroscopy mapping images of oxygen, boron, and nitrogen showing the heterostructure corresponding to the yellow box in (B). (D) Conductance trajectories of the excitatory postsynaptic current and inhibitory postsynaptic current when applying pulses with 10 ms width and 2 V amplitude to the WCT. Reprinted with permission from (Seo et al., 2018). Copyright 2018 Springer Nature. (E) Switching performance of a GMG device at different temperatures. The arrows indicate the switching direction. (F) *In situ* cross-sectional STEM images and corresponding EDS line profiles of a single GMG device in the pristine (left), ON state (center), and OFF state (right). EDS line profiles obtained from the region of green arrows in STEM images. Reprinted with permission from (Wang et al., 2018). Copyright 2018 Springer Nature.

Seo et al. demonstrated a vdW ONS device by implementing synaptic and optical-sensing functions on h-BN/WSe_2_ heterostructure through the charge trapping/detrapping process, as depicted in Figure 5A (Seo et al., 2018). To implement a synaptic function to their device, the top surface of h-BN was treated by O_2_ plasma for adjustment of the conductivity of the WSe_2_ channel on the h-BN, resulting in a WCL. Cross-sectional TEM analysis was performed to investigate the structural and atomic compositions of the WSe_2_/WCL/h-BN region, as shown in Figure 5B. The WCL layer formed by O_2_ plasma treatment showed the oxygen (O) and boron (B) signals in the electron energy loss spectroscopy analysis (Figure 5C), indicating that the WCL layer consisted of the oxidized boron transformed from the oxidation of h-BN. Figure 5D represents the synaptic functionality with excitatory (V > 0) and inhibitory (V < 0) spikes that are controlled by the trapped charge in the WCL. The device operated with low voltage spike amplitude of 0.3 V and low energy consumption of only 66 fJ per spike. Moreover, they also demonstrated diverse synaptic functions, including LTP, LTD, and STDP dynamics showing excellent linearity with a nonlinearity of 1.4/1.4 for weight increase/decrease.

By utilizing ionic migration, Wang et al. demonstrated robust memristors with 2D vdW heterostructure, which consists entirely of the sandwiched 2D layered materials of graphene/MoS_2-x_O_x_/graphene (GMG) (Wang et al., 2018). As seen in Figure 5E, the GMG memristive device maintains the resistive switching property at high temperature up to 340°C, which is higher than the low operating temperature limit of 200°C for conventional oxides (Chen et al., 2012; Lee et al., 2008). Such high-temperature robustness of 2D vdW heterostructure memristors is attributed to the atomically sharp interface between the graphene and MoS_2-x_O_x_ layer as well as the high-temperature structural stability of MoS_2-x_O_x_. The resistive switching mechanism of the device was investigated by *in situ* STEM and EDS analysis. As shown in the STEM images and corresponding EDS profiles in Figure 5F, sulfur and oxygen atoms were observed to migrate during the switching. The sulfur ions in the channel region were reduced in the ON-state, and the oxygen ions in the channel region were increased after OFF-state, indicating that oxygen ions migrated into the channel region and filled sulfur vacancies during the RESET process. Furthermore, the GMG devices on the PI substrate showed high flexibility and mechanical endurance during the bending test of >1,000 cycles, indicating a great potential for flexible electronic devices. 2D heterostructures exhibit intrinsic advantages uniquely suitable for neuromorphic applications. These include the preservation of the structural, chemical, and electrical qualities of individual 2D components circumventing the lattice match constraint of conventional thin film materials. For instance, in Figure 5B, the h-BN is heterogeneously integrated with the WSe_2_ layer forming the WCL, enabling to modulate a number of interfacial traps and achieve synaptic functionalities unaffected by the interfacial defects between each 2D material. Moreover, the top and bottom graphene electrodes are integrated with the MoS_2_ layer, which allows for the high-temperature operation of the memristive devices and chemical stability coupled with large Young's under mechanical deformation owing to their well-preserved thermal modulus. A comprehensive summary of recent studies on 2D vdW heterostructures with respect to their respective synaptic characteristics and dimensions are presented in Table 2.

**Table 2:** Neuromorphic 2D vdW Heterostructures Devices Based on 2D Materials

Heterostructure Materials	Thickness (nm)	Electrode	Growth/Fabrication Method	Stacking Method	Working Principle	Retention Time (s)	Endurance (Cycle)	Power Consumption/Switching Voltage	Device	Ref.
Gr/MoS_2_	MoS_2_: 21	Ni (top)	CVD	Wet transfer	Grain boundary ion migration	–	40	2.8–2.9 V	Artificial neuron	(Kalita et al., 2019)
		Gr (bottom)								
Gr/MoS_2_	NA	Ni/Au (top)	CVD	Wet transfer	Conductive filament	10^4^	100	1.5 V	Synaptic memristor	(Krishnaprasad et al., 2019)
		Gr (bottom)								
Gr/MoS_2-x_O_x_/Gr	Gr: 8	Au (top)	Exfoliation	PVA transfer	Ion migration	10^5^	10^7^	–	Flexible electronic device	(Wang et al., 2018)
	MoS_2-x_O_x_: 40	Au (bottom)								
MoS_2_/h-BN/Gr/h-BN	MoS_2_: 6	Cr/Au (top)	Exfoliation	PVA transfer	Charge trapping/detrapping	–	100	64 pJ	Human memory system	(Chen et al., 2019a)
	h-BN: 7, 15	Si (bottom gate)								
Gr/h-BN/MoS_2_	h-BN: 3.5–12	Cr/Au	Exfoliation	PVA transfer	Charge trapping/detrapping	10^5^	10^5^	4.8 V	Flexible floating gate memories	(Vu et al., 2016)
Gr/WSe_2_	WSe_2_: 30–60	Cr/Pd/Au (top)	Exfoliation	Polymer-assisted transfer method	Conductive filament	10^3^	10^3^	0.2 V	Artificial synaptic barristor	(Huh et al., 2018)
		Ag (bottom)								
h-BN/MoS_2_/h-BN/Gr or MoS_2_	h-BN: 15–20	Cr/Au (Top)	Exfoliation	Polymer-assisted transfer method	Charge trapping/detrapping	10^4^	300	–	Multilevel optical memory	(Kim et al., 2019c)
	MoS_2_: 3–17	P^++^-Si (bottom)								
h-BN/MoS_2_/h-BN/MoS_2_	MoS_2_: 10–14	Cr/Au (top)	Exfoliation	Polymer-assisted transfer method	Charge trapping/detrapping	10^4^	–	–	Flash memory devices	(Yi et al., 2018)
	h-BN: 10–34	Au, Al, Pd (bottom)								
h-BN/WSe_2_	–	Ti, Au (control)	Exfoliation	Residue-free transfer method	Charge trapping/detrapping	–	300	66 fJ at 0.3 V pulse	Optic-neural synaptic device	(Seo et al., 2018)
		Pt/Au (synaptic)								
Gr/h-BN/MoS_2_	h-BN: 5–7	Cr/Au (top)	Exfoliation	Dry-transfer method	Charge trapping/detrapping	–	20	5 fJ	Floating gate memory	(Paul et al., 2019)
		P^++^-Si (bottom)								
BP/SnSe_2_	BP: 6 SnSe_2_: 100	Au	Exfoliation	Transfer method	Charge trapping/detrapping	–	–	1 fJ for SET, 100 aJ for RESET	Synaptic memristor	(Tian et al., 2017a)
MoS_2_/PTCDA	Total: 10	Au (top)	Exfoliation	Direct growth	Charge trapping/detrapping	–	–	10 pJ	Artificial synapse	(Wang et al., 2019b)
		n^++^-Si (bottom)								

Gr, graphene; PVA, polyvinyl alcohol; PTCDA, perylene-3,4,9,10-tetracarboxylic dianhydride.

## 2D Materials-Based Neuromorphic Device Applications

### 2D-Based Electronic Synapses

Graphene has been exploited as electrodes in oxide-based memristors owing to its semi-metallic and gapless nature (Bai et al., 2015; Chakrabarti et al., 2014; Lee et al., 2015; Ni et al., 2018; Tian et al., 2013, 2017b). The incorporation of graphene into device components can help decrease the programming current due to its high out-of-plane resistance in vertical electrode geometries. Chakrabarti et al. reported sub-μA current operation and low RESET power in graphene/insulator/graphene stacks employing CVD-graphene as both bottom/top electrodes and TiO_x_/Al_2_O_3_/TiO_2_ layers as an insulator layer. It was observed that graphene acted as an oxygen reservoir for the formation of filaments composed of oxygen ions based on the weak physisorption of oxygen ions with the graphene, which enables facile diffusion of oxygen ions (Chakrabarti et al., 2014). Another noteworthy study by Lee et al. demonstrated a HfO_x_ memristor with a CVD-graphene edge electrode, which yielded low variability in SET and RESET voltages with a reduced energy consumption of around ∼230 fJ. The ultra-thin edge side of the 3-nm graphene layer directly interfaced with the HfO_x_ memristor allows a much stronger electric field lowering the activation energy for oxygen migration, and thus power consumption, compared with other electrode materials (e.g., titanium nitride) (Lee et al., 2015). However, due to its intrinsic zero-band-gap feature, the application of graphene has been limited to mostly electrodes, thus other types of 2D materials have been further explored for more versatile applications in synapses.

Various conduction mechanisms have been attributed to the observation of synaptic characteristics such as plasticity, multiple conductance states, and weight updates. The underlying principles responsible for the conduction mechanisms are determined by carrier transport characteristics, e.g., filamentary, defect- or interface-mediated transports, trap-assisted tunneling, and phase transitions, which have also been observed in conventional oxides- or PCM-based synaptic devices. 2D materials-based synaptic devices often exhibit high linearity in the weight update superior to the conventional ones, which is attributed to their intrinsically anisotropic non-layered vdW structure unlike structurally isotropic materials.

**Figure 6:**
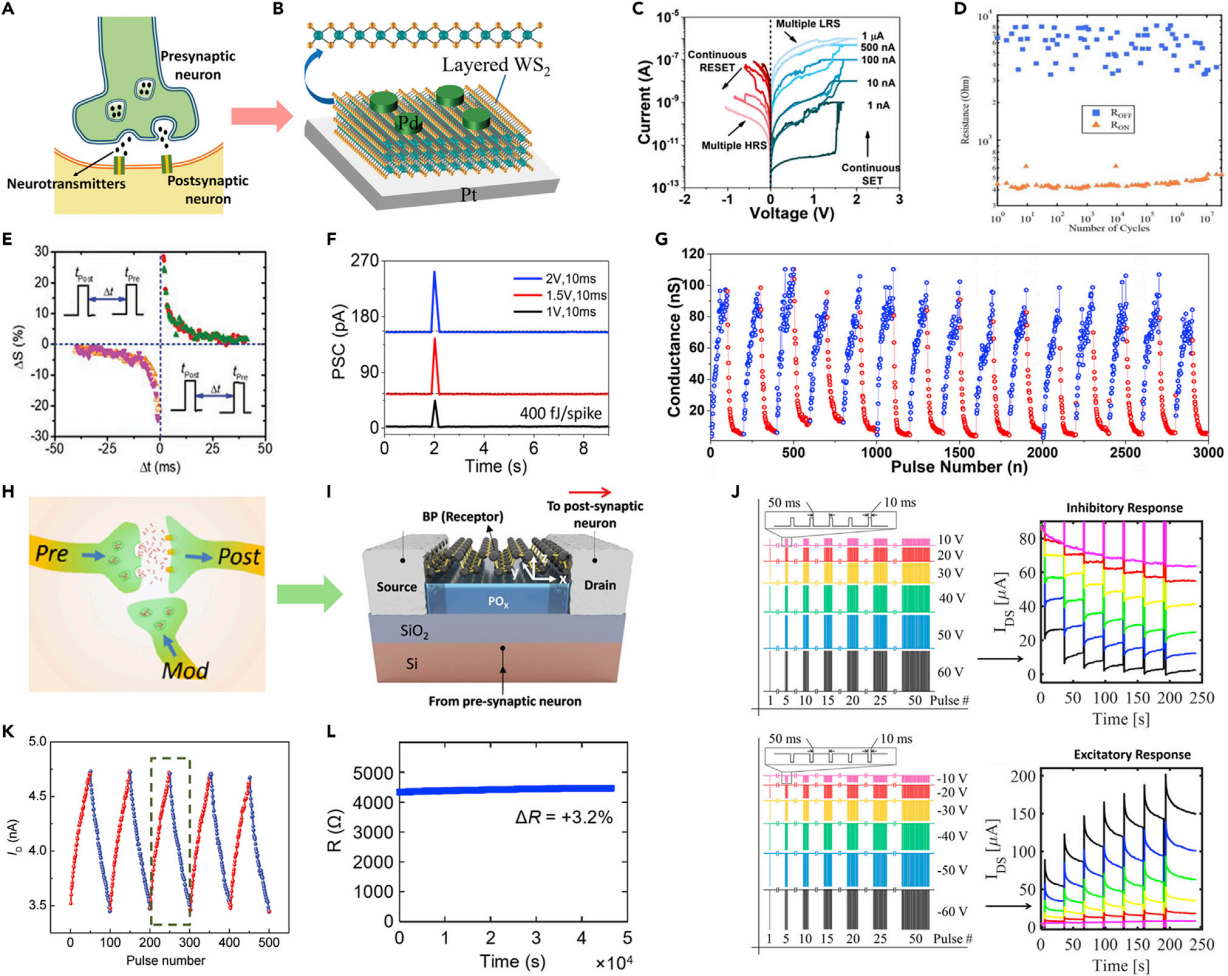
2D-Based Electronic Synapses (A) Schematic representation of a biological synapse. (B) Synaptic device realized by a 2-terminal memristor device with 2D WS_2_ as the switching medium in a Pd/WS_2_/Pt stack. Reprinted with permission from (Yan et al., 2019b). Copyright 2019 WILEY-VCH Verlag GmbH & Co. KGaA, Weinheim. (C) Multiple conductance states corresponding to the synaptic weight states exhibited by the Ni/MoS_2_/graphene synapse under DC bias with the lowest programming current of 1 nA. Reprinted with permission from (Krishnaprasad et al., 2019). Copyright 2019 AIP Publishing. (D) Robustness of the 2D memristors with active MoS_2_ medium and graphene electrodes showing sustained switching between HRS and LRS for 10^7^ cycles. Reprinted with permission from (Wang et al., 2018). Copyright 2018 Springer Nature. (E) Demonstration of STDP learning rule in a Ag/ZnO/WS_2_/Al synapse indicating the viability of 2D material-based synapses for unsupervised learning. Reprinted with permission from (Kumar et al., 2019). Copyright 2019 WILEY-VCH Verlag GmbH & Co. KGaA, Weinheim. (F) Low energy consumption of ∼400 fJ recorded in a 2D perovskite-based synapse, which is one order of magnitude higher than that of the biological synapse. Reprinted with permission from (Tian et al., 2017c). Copyright 2017 American Chemical Society. (G) Near-linear weight update observed in a Ni/MoS_2_/graphene synapse with an NLF of 0.276 along potentiation sustained for 15 cycles. Reprinted with permission from (Krishnaprasad et al., 2019). Copyright 2019 AIP Publishing. (H) Biological synapse schematic with a modulatory input required for implementing heteroplasticity. Reprinted with permission from (He et al., 2020). Copyright 2020 American Chemical Society. (I) Schematic illustration demonstrating a synapse utilizing a BP-based FET device. Reprinted with permission from (Tian et al., 2016). Copyright 2016 WILEY-VCH Verlag GmbH & Co. KGaA, Weinheim. (J) Implementation of both inhibitory and excitatory synapse with their corresponding inputs demonstrated using a back-gated MoS_2_ FET by utilizing the gate terminal. Reprinted with permission from (Arnold et al., 2017). Copyright 2017 American Chemical Society. (K) Weight update observed in ionic gate MoO_3_ FET. Reprinted with permission from (Yang et al., 2017). Copyright 2017 WILEY-VCH Verlag GmbH & Co. KGaA, Weinheim. (L) Data retention of > 10^4^ s observed in graphene FET intercalated with Li^+^. Reprinted with permission from (Sharbati et al., 2018). Copyright 2018 WILEY-VCH Verlag GmbH & Co. KGaA, Weinheim.

Various semiconducting 2D materials have been successfully integrated as active layers for synaptic devices (Figures 6A and 6B) (Yan et al., 2019b). Particularly, 2D TMDs employed as active switching media enabled the lowering of programming current down to 1 nA (Figure 6C) (Krishnaprasad et al., 2019) as well as achieving good endurance, as shown in Figure 6D (Wang et al., 2018). Initial efforts were focused on demonstrating memristive behaviors using a composite of graphene oxide and MoS_2_ as the active layer (Yin et al., 2013). For instance, vertical memristive devices were realized by stacking up exfoliated MoS_2_ nanosheets (Cheng et al., 2016) with graphene oxide in composite structures (Choudhary et al., 2019; Shin et al., 2016). The high-quality interface rendered by these 2D materials enabled a GMG device to exhibit high retention (>10^4^ s), high pulsed endurance (10^7^ cycles), high switching speed (∼100 ns), and retained performance having withstood over 1,200 bending cycles (Wang et al., 2018). Additionally, the low energy consumption of ∼1 fJ was reported in an Ag/BNO_x_/graphene stack (Zhao et al., 2017). A variety of memristors have been successfully fabricated with CVD-grown 2D materials, although the prevalent issue of their limited scalability and uniformity hinders their large-scale realization (Ge et al., 2018; Qian et al., 2016). However, it should be noted that none of these approaches successfully demonstrated synaptic characteristics despite excellent features in other aspects, i.e., retention, endurance, and power consumption. 2D materials-based synapses have been mainly explored in two different types of device configurations, such as two- and three-terminal devices. Hereafter, major emphasis will be placed on reviewing the synthesis processes of 2D materials for synaptic devices and their device performance metrics.

#### Two-Terminal Devices

Plasticity is one of the critical features in synapses governed by their learning rules and is broadly classified into two categories, i.e., homosynaptic and heterosynaptic plasticity. Homosynaptic plasticity is governed by the Hebbian learning rule, where the synaptic efficacy is determined by the presynaptic and postsynaptic inputs of the synapse involved in the process. This plasticity is associated with memory and learning. STDP learning is associated with the unsupervised learning of the ANNs. A representative plot of the STDP, as demonstrated in a two-terminal ZnO/WS_2_/Al synaptic device, is shown in Figure 6E (Kumar et al., 2019). Another study on memristive switching in Ag/MoO_x_/MoS_2_/Ag vertical stacks reported their synaptic plasticity characteristics like STP and LTP, achieved at an operating voltage of 0.1 V (Bessonov et al., 2015). Similarly, synaptic memristors based on exfoliated MoS_2_ flakes were reported (Li et al., 2018; Yin et al., 2019a). For instance, Yin et al. studied synaptic behaviors in a lateral Ag/MoS_2_/Pt device, where the resistive switching was attributed to the formation of filaments composed of Ag nanoparticles. The aforementioned synaptic devices, characterized by weight updates, were observed to follow the learning curve of *Drosophila*. The filamentary mechanism was also observed in a two-terminal vertical memristor that employed graphene/Au as top/bottom electrodes and 2D perovskite as the active switching medium (Tian et al., 2017c). This device showed synaptic characteristics of STP and LTP with an energy consumption of ∼400 fJ (Figure 6F).

In contrast to the filament formation mechanism, phase transition behaviors inherent to certain 2D materials are attributed to be responsible for resistive switching in many cases (Zhang et al., 2019; Zhu et al., 2019). 2D MoS_2_ flakes initially in 2H phase can be transformed into metallic 1T phase by Li^+^ intercalation, which can lead to a reversible conductance change tuned by an electric field; i.e., 1T phase (SET) from/to 2H phase (RESET) (Zhu et al., 2019). Similarly, Zhang et al. reported a Ti/Au/MoTe_2_/Ti/Ni memristor with high retention of 10^5^ s, where the weight update characteristic was induced by the phase transition of MoTe_2_ via electric field (Zhang et al., 2019). Intrinsic structural defects such as chalcogen vacancies in TMDs also contribute to the resistive switching (Li et al., 2018; Yan et al., 2019b). The memristive synapse of Pd/WS_2_/Pt showed the small energy consumption of ∼300 and ∼125.6 fJ in SET and RESET operations, respectively, with a switching speed of 500 ns, enabled through vacancy migrations. These devices also exhibited the STDP learning rule required for unsupervised learning. Furthermore, synaptic characteristics such as pulse paired facilitation (PPF), STP, LTP, and STDP were observed in graphene/WSe_2_/WO_3-x_/Ag (Huh et al., 2018) and ZnO/WS_2_/Al memristive devices (Kumar et al., 2019). However, all the aforementioned device demonstrations employed mechanically exfoliated 2D flakes of small dimensions. To realize ANNs, large-scale synthesis (e.g., CVD-growth) of 2D TMDs is imperative to employ them as switching media for synaptic operation (Krishnaprasad et al., 2019; Shi et al., 2018; Xu et al., 2019). Additionally, the trajectory of the weight update plays a critical role in determining the viability of new material systems for ANN applications. In that effort, 2D materials show the coveted near-linear weight update with identical pulses. In the aforementioned report by Krishnaprasad et al., near-linear weight update was observed in Au/Ni/MoS_2_/graphene devices with identical programming pulses, leading to the nonlinearity factor (NLF) of 0.276 along potentiation. These devices also exhibited other synaptic characteristics like STP, LTP, and STDP, shown in Figure 6G. In this device, a large-area CVD-grown MoS_2_ acted as the switching medium on the graphene bottom electrode. Although the exact mechanism responsible for such superior linearity in 2D materials-based synapses remains unclarified at this point, it is proposed to be likely a result of the interplay of filamentary and interface-mediated transports. In conventional oxide-based memristors, improved linearity in the weight update is observed when the switching is driven by the interface-mediated transports. In the case of Au/Ni/MoS_2_/graphene devices, there exists an additional variable of MoS_2_/graphene interface in addition to the anticipated filamentary mechanism. This 2D/2D interface should exhibit the Schottky barrier that further modulates their diffusion kinetics at the bottom electrode. The verification of the proposed transport model needs further investigations (Ge et al., 2018; Krishnaprasad et al., 2019). Additionally, an ultra-low switching voltage of 100 mV was achieved in metal-organic CVD (MOCVD)-grown MoS_2_ with a device structure of Cu/MoS_2_/Au (Xu et al., 2019). From the performance metrics perspective, not all the key features emphasized in this review article have yet been achieved by a single device in any of these previous reports. However, it is worth noting that the current findings of ∼fJ power consumption, near-linear weight update with identical programming pulses, high retention, and endurance on the flexible platform along with large-scale synthesis processes present promising prospects for 2D materials in neuromorphic applications.

#### Three-Terminal Devices

In this section, we review artificial synapses reported employing three-terminal devices. For heterosynaptic plasticity, synaptic devices are required to be in three-terminal geometries, which are more suitable for accommodating the LTP with multiple synapses to maintain strong and efficient synaptic association, demanded in various applications such as sound localization (Das et al., 2019; Sun et al., 2018). In the three-terminal devices that emulate heterosynaptic plasticity, the third input is utilized as the modulatory one (Figure 6H) as realized in FETs, which can be used as logic gates by emulating a biological process of dendritic integration (He et al., 2020).

Three-terminal FETs can emulate the dual modes of the synaptic behavior, inhibitory and excitatory, operated by the trapping/detrapping of carriers (Arnold et al., 2017; Tian et al., 2015, 2016, 2017a). For instance, the FET of exfoliated BP flake shown in Figure 6I (Tian et al., 2016) exhibited synaptic characteristics such as STDP and PPF availing the ambipolarity of BP, which induced the charge trapping/detrapping by forming phosphorus oxide (PO_x_). Furthermore, the inhibitory and excitatory synaptic behaviors were observed in the back-gated MoS_2_ FETs manifested by their large I-V hysteresis, as shown in Figure 6J (Arnold et al., 2017). Interestingly, back-gated graphene FETs exhibited the tunable synaptic plasticity (Bai et al., 2015) as well as the emulation of the synaptic behavior by capacitive gating (Guo et al., 2019; Xie et al., 2018b; Yang et al., 2017). In these approaches, the ionic liquid gating in the MoO_3_ flake of ∼12 nm thickness induces essential synaptic characteristics of PPF, STP, and LTP. The weight updates observed in these devices were near-linear and symmetric, as shown in Figure 6K, and the power consumption was observed to be ∼9.6 pJ (Yang et al., 2017). Energy consumption was further decreased in a MoS_2_ FET using a graphene floating gate (Paul et al., 2019), which demonstrated STDP with a remarkable endurance of 10^5^ cycles and energy dissipation of ∼5 fJ. Recently, it was reported that a dual-gated MoS_2_ FET exhibited tunable synaptic plasticity and high linearity of NLF = 0.5 during potentiation with identical input pulses (He et al., 2020). Along with the results employing exfoliated 2D materials, CVD-grown MoS_2_ was also exploited in achieving a six-terminal memtransistor. This device utilized the variable conductance states obtained by Schottky barrier tunneling and achieved a long retention time of the order of a decade while presenting non-linear updates (Sangwan et al., 2018). Meanwhile, linear updates were obtained in graphene FETs intercalated by Li^+^, which showed excellent retention of >10 h and STDP learning rule (Figure 6L) (Sharbati et al., 2018). These devices exhibited conductance states of >250 with an energy consumption of <500 fJ, suggesting viability for ultra-low-power neuromorphic applications.

### 2D-Based Optoelectronic Synapses

Another category of synapse that has been widely explored is the optoelectronic synapse. The synaptic devices emulate biological optical synaptic characteristics by using the response to light of a wide range of wavelengths. The current semiconductor technology for image sensors is categorized into two types, i.e., charge-coupled devices and active pixel sensors (Bigas et al., 2006). These devices capture image information from the environment and further convert it into digital format, with the accumulated data subsequently transferred to a computing unit. In this case, a huge amount of the obtained data is redundant, which causes latency and a power consumption increase. Therefore, the data movement should be optimized by utilizing an analog device that can simultaneously function as a computing and memory unit with a response to optical stimuli. In this regard, optoelectronic synapses are being explored in pursuing such devices that employ light-sensitive materials that can exhibit potentiation under optical stimulation. Furthermore, these devices should essentially possess synaptic characteristics of STP, LTP, PPF, STDP, and data retention.

Much progress has been made in emulating biological optical synapse characteristics exploiting the photosensitivity characteristics of 2D materials. The main working principle of these optoelectronic devices is the trapping and detrapping of the photo-generated carriers. This charge trapping/detrapping can happen due to many factors, including intrinsic defects of the channel material, trap sites in the channel/gate oxide interface, and the absorption of oxygen ions from the environment (Late et al., 2012). The optoelectronic synapses are categorized into three different types of devices depending on their components. (1) Intrinsic 2D materials are used as their conducting channels. (2) Hybrid channels are used, incorporating nanoparticles into the 2D semiconductor channels (Ni et al., 2018). These nanoparticles are sensitive to a particular wavelength of the visible light and can create photo-generated carriers upon illumination. (3) Devices composed of vdW heterostructures. The optical neural network can be developed using optoelectronic synapses for pattern recognition and other neuromorphic applications. Here, we summarize the recent research progress on 2D materials-based optoelectronic synapses.

#### Intrinsic Channel 2D Optoelectronic Synapses

**Figure 7:**
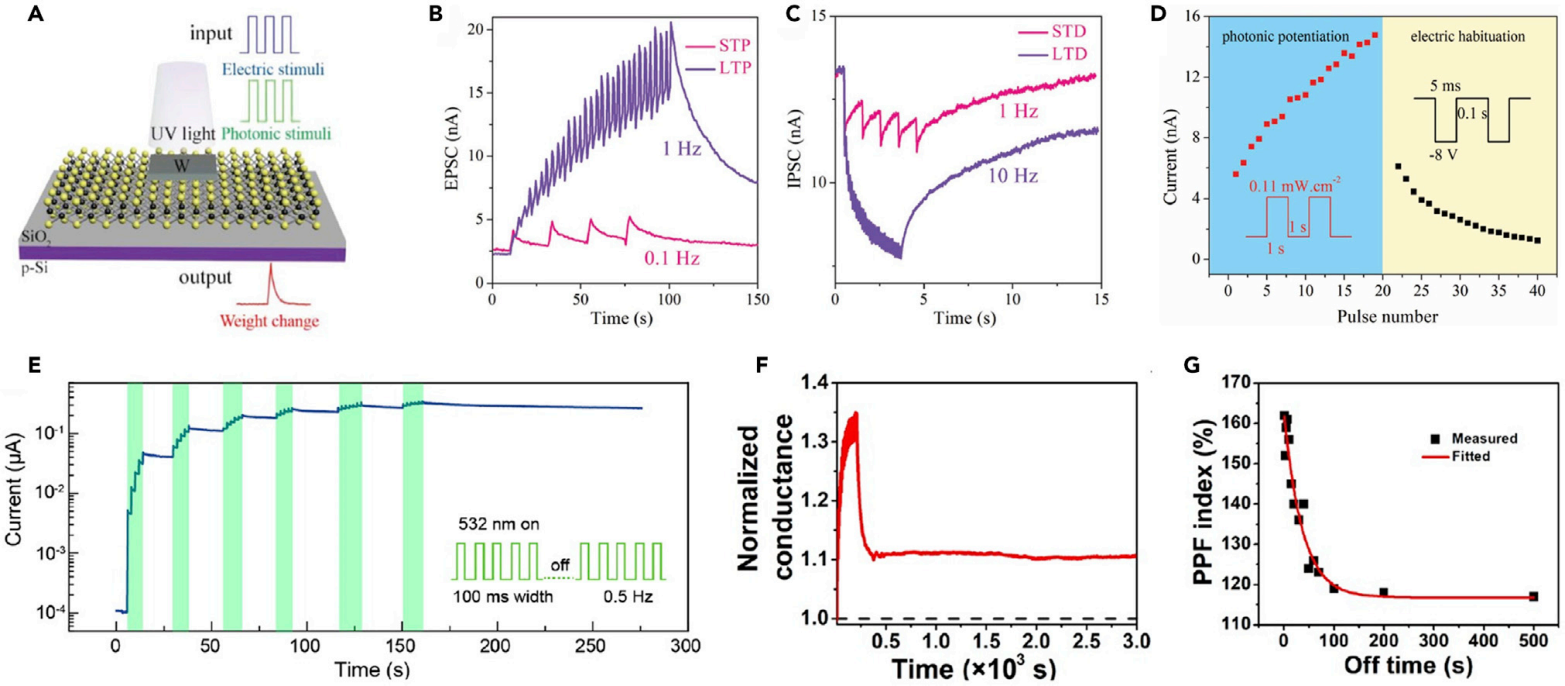
2D-Based Optoelectronic Synapses (A) Schematic illustration of an optoelectronic synapse using 2D material. (B) STP and LTP behavior emulated by photonic stimuli (STP: width = 1 s, f = 0.1 Hz, LTP: width = 1 s, f = 1 Hz). (C) STD and LTD behavior emulated by electrical stimuli (STD: width = 5 ms, f = 1 Hz, LTD: width = 5 ms, f = 10 Hz, amplitude was fixed at −8 V). (D) Photonic potentiation and electric habituation. Reprinted with permission from (He et al., 2018). Copyright 2018 WILEY-VCH Verlag GmbH & Co. KGaA, Weinheim. (E) Demonstration of multilevel channel conductance by the application of a train of light pulses. Reprinted with permission from (Luo et al., 2020). Copyright 2020 American Chemical Society. (F) Retention for 3 × 10^3^ s after LTP as a response to 20 light pulses. (G) PPF index for varying intervals between two consecutive pulses at different wavelengths. Reprinted with permission from (Pradhan et al., 2020). © The Authors, some rights reserved; exclusive licensee American Association for the Advancement of Science. Distributed under a Creative Commons Attribution NonCommercial License 4.0 (CC BY-NC) http://creativecommons.org/licenses/by-nc/4.0/

He et al. fabricated ultra-thin optoelectronic synapses using CVD-grown monolayer MoS_2_ (0.65 nm in thickness) as the conducting channel on p-Si (schematic illustration is shown in Figure 7A). Upon light illumination, the photo-generated electrons and holes are separated by the built-in electric field across the n-MoS_2_/p-Si heterojunction. In this device, synaptic neuromorphic functions, such as short-term memory, long-term memory (Figures 7B and 7C), and PPF, were successfully emulated along with optical potentiation and electric habituation behaviors (Figure 7D). The conductance of monolayer MoS_2_ is considered as the synaptic weight that can be modulated by the synaptic spikes of photonic and electric stimuli. The increase of the conductance by the optical stimulus is the potentiation, whereas the decrease of the conductance by the electric stimulus is the habituation of the synaptic strength (He et al., 2018). Ahmed et al. demonstrated similar optoelectronic characteristics in a few-layer BP (9.2 nm in thickness). By absorbing oxygen from the environment, the exfoliated BP flakes were oxidized into PO_x_ layers, which played an important role in defining photoresponse in the synaptic devices. The excitatory and inhibitory action potentials were initiated directly by optical stimulation. The inherently repeatable photoconductivity of BP under UV and visible excitation wavelengths was deployed to emulate various synaptic functions; these include short-term memory to long-term memory, potentiation, inhibition, pulsed-pair facilitation, spatiotemporally correlated dynamic logic, and Hebbian and associative learning for decision-making. Under the illumination of 280 nm optical stimulus, the few-layer BP flakes exhibited positive photocurrent corresponding to excitatory, whereas those under 365 nm illumination displayed negative photocurrent corresponding to inhibitory postsynaptic currents (Ahmed et al., 2019). Additionally, Kim et al. demonstrated germanium (Ge)-gated MoS_2_ phototransistors for optical sensing and synaptic operation, which can respond to visible and infrared light. Germanium, with its narrow band gap, was employed as a back-gate electrode of the phototransistor to detect infrared light, and multilayer MoS_2_ flakes were integrated onto it by a mechanical exfoliation method. Under an infrared light illumination (λ = 1550 nm), the MoS_2_ phototransistor showed a positive shift of V_th_ from −0.553 to −0.312 V and a reduction of I_D_ by a factor of 35 at V_G_ = −0.5 V and V_D_ = −0.5 V. Under visible light illumination (λ = 520 and 655 nm), the Ge-gated MoS_2_ phototransistor showed an increment in I_D_ by a factor of >1,000 at V_G_ = −0.5 V and V_D_ = −0.5 V. The input gate voltage pulses of −30 V and 15 V were applied to mimic the excitatory and inhibitory synaptic transmissions, respectively (Kim et al., 2019b). By using dielectric engineering, Luo et al. demonstrated 2D optoelectronic synapses composed of few-layer WS_2_ (conducting channel) and PbZr_0.2_Ti_0.8_O_3_ (PZT) thin film (ferroelectric gate dielectric). The conductance of the WS_2_ channel was modulated by the switchable and permanent polarization of PZT. This memristive transistor successfully mimicking the neuromorphic synaptic properties, such as STP and LTP, was used as the optical information-driven long- and short-term memory. The LTP and LTD were achieved by applying optical pulses (λ = 532 nm, P = 10 μW) and electrical pulses of amplitude −3.5 V, respectively. The multilevel conductance states (Figure 7E) were produced by applying multiple trains of pulses. When a voltage was applied to the PZT through the WS_2_, PZT forms the upward or downward ferroelectric domains underneath the WS_2_, leading to a positive or negative polarization to the surface areas, respectively. Therefore, it results in the accumulation or depletion of charges in the adjacent WS_2_ channel (Luo et al., 2020). Furthermore, Zhang et al. demonstrated a charge-trapping memory synaptic device based on 2D MoS_2_ (2.9 nm in thickness) and high-*k* Ta_2_O_5_–TiO_2_ (TTO) gate dielectric. The charge trapping states can be modulated by changing the sweep range of the back-gate voltage (V_BG_). The device successfully achieved distinct memory states upon both optical and electrical pulses. For a negative V_BG_, electrons tunnel through the 4-nm-thick Al_2_O_3_ barrier and are trapped in the TTO layer. This trapping mechanism enables the device to mimic optical synaptic properties (Zhang et al., 2020). Similarly, John et al. demonstrated a three-terminal MoS_2_-based optoelectronic synapse device via multi-gating approaches emulating various synaptic characteristics. The device was operated via the trapping-detrapping of electrons at the semiconducting channel as well as the migration-relaxation of ions in the gate dielectric (John et al., 2018).

#### Hybrid Nanoparticles/2D Semiconductor Optoelectronic Synapses

Pradhan et al. demonstrated an optoelectronic synapse in a hybrid nanoparticles/2D semiconductor configuration by using monolayer graphene and perovskite quantum dots (PQDs) grown from the lattice of the graphene. In this approach, the graphene and the PQDs acted as the carrier transport channel and the photo-absorbing layer, respectively, and the photogeneration efficiency of methylammonium lead bromide PQDs was studied. The PQDs generated photo-excited free charge carriers upon absorbing photon energy higher than their band gap. The device showed a photoresponsivity of 1.4 × 108 A W^−1^ at 430 nm and a specific detectivity (D∗) of 4.72 × 10^15^ Jones. It also showed STP, LTP, LTD, data retention of 3 × 10^3^ s (Figure 7F), PPF (Figure 7G), and other essential synaptic characteristics with a low energy consumption of 36.75 pJ per spike (Pradhan et al., 2020). Ni et al. reported a different synaptic device using 2D WSe_2_ with B-doped Si nanocrystals (NCs). This Si-NC/WSe_2_ device showed comprehensive synaptic characteristics in a broad spectral region from near-infrared (NIR) to UV, which stemmed from the strong broadband optical absorption of B-doped Si-NCs. The change in device conductance was shown as a response to a 1342-nm laser spike originating from the B-doping-induced band-tail optical absorption of Si NCs, and the subsequent transfer of the photo-generated holes from Si NCs to WSe_2_ (Ni et al., 2018). In an additional demonstration, Qin et al. devised an optical neuromorphic device based on a hybrid film combining graphene and single-walled carbon nanotubes (SWNTs). This device showed optoelectronic synapse features due to its charge trap-rich interface between the hybrid film and the substrate. When a negative bias was applied at the gate, photo-generated holes in graphene and SWNTs were partially trapped into the trap sites at the interface, which resulted in a long-term stable photo-gating effect even after the incident light was switched off due to the high trapping energy barrier (Qin et al., 2017). Another demonstration of optoelectronic resistive random access memory (ORRAM) synaptic device by Zhou et al. was shown using a two-terminal structure of Pd/MoO_x_/ITO. Under UV light illumination of 365 nm, the device was switched to LRS, which was retained even after the illumination was turned off. A variety of image device functionalities, including image sensing and memorization, as well as a real-time image preprocessing, such as image contrast enhancement, were demonstrated with these ORRAM arrays (Zhou et al., 2019).

#### vdW Heterostructures Optoelectronic Synapses

**Figure 8:**
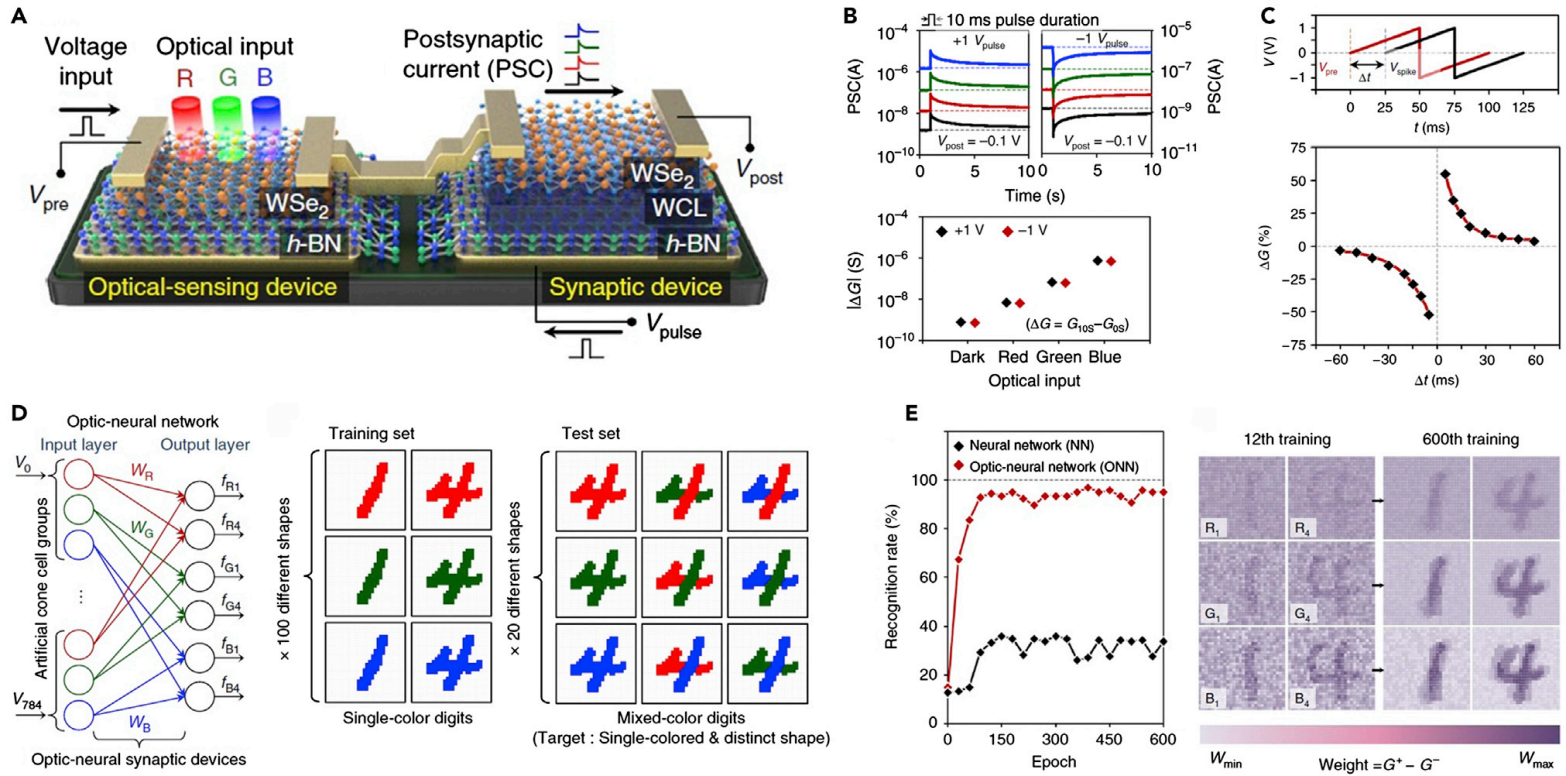
vdW Heterostructure Optoelectronic Synapses (A) Schematic illustration of an h-BN/WSe_2_ synaptic device integrated with h-BN/WSe_2_ photodetector. (B) Postsynaptic current characteristics and extracted conductance changing under different light conditions. (C) STDP behavior obtained from the synaptic device. (D) ONN for recognition of 28 × 28 RGB-colored images. (E) (Left) Training and the testing datasets consisting of single-colored and color-mixed numeric pattern images. (Right) Weight mapping images after the 12th and 600th training epoch. Reprinted with permission from (Seo et al., 2018). Copyright 2018 Springer Nature.

For vdW heterostructures-based optoelectronic synapse devices, Wang et al. demonstrated an h-BN-encapsulated MoS_2_ optoelectronic synapse fabricated on aluminum oxide (AlO_x_)/Si substrate. The thickness of the exfoliated MoS_2_ was 1.7 nm and that of the h-BN was approximately 7 nm. Due to the charge-trapping between the MoS_2_ and AlO_x_ interface, this device exhibited basic synaptic functions. The h-BN encapsulation afforded stability to the device, achieving various conductance states with varying V_G_ upon a single laser pulse (λ = 532 nm). Therefore, continuous laser pulses can emulate STP and LTP of the device, and PPF characteristics were also mimicked (Wang et al., 2019c). Additionally, Tian et al. demonstrated a heterostructure conducting channel with layered 2D perovskite materials sandwiched between top/bottom graphene layers, with the bottom one serving as a channel with ambipolar transport characteristics. The light absorption by the 2D perovskite mobilized I^−^ ions (or holes), leading to their transport by the applied electric field. When the continuous light pulses (λ = 520 nm) were applied at −500 mV (or 500 mV), the device was potentiated (or depressed). The STP and LTP of the device are attributed to the electron-hole pairs and the I^−^ ions, respectively (Tian et al., 2018). Last, Seo et al. demonstrated a vdW heterostructure-based optoelectronic synapse by integrating an h-BN/WSe_2_ synaptic device with an h-BN/WSe_2_ photodetector (Figure 8A). The optical synapse showed distinct synaptic weight changes for LTP/LTD in response to light of different wavelengths (red, green, and blue) while maintaining the curved shape related to nonlinearity, as demonstrated in postsynaptic current measurement (Figure 8B). The synaptic operation of the device was based on the trapping and detrapping of electrons in the WCL and the LTP and LTD curves were obtained at 0.3 V under various light illuminations. The STDP behavior of the synaptic device was also confirmed (Figure 8C), indicating that this device can be applied to spiking neural networks (SNNs) with the STDP learning algorithm. An artificial ONN was explored (Figure 8D) assisted by a simple perceptron network model and was applied to accomplish colored and color-mixed pattern recognition tasks. The recognition rate for the ONN exceeded 90% after the 50th epoch (Figure 8E), whereas the recognition rate for the neural network, which was composed of synaptic devices without the optical sensing function, remained below 40% (Seo et al., 2018).

### 2D-Based Neurons

The past few years have witnessed considerable research efforts in achieving 2D materials-based electronic and optoelectronic synapses. Despite the projected opportunities of 2D materials for compact, scalable, and energy-efficient artificial neurons, their experimental exploration is still in its infancy. This limitation is due to the complex and challenging nature of emulating the transient switching with rich dynamics as well as processing data at the temporal or frequency domain similar to the biological neuron (Hao et al., 2020). In neural networks, the artificial neurons receive, process, and transmit signals through ionic movements. In a lipid bilayer membrane, neurons contain leaky ion channels and modulate the movements of ions (Na^+^, K^+^) between the extracellular and intracellular fluids. With the incoming signals through synapses, the membrane potential changes due to the variation of the ion concentration between these two fluids. Once the membrane potential reaches a threshold value, the ions start to flow through the leaky ion channels to transmit the signals. Afterward, the membrane potential returns to the equilibrium state. These neuronal characteristics are explained by various models such as integrate-and-fire (IF), leaky integrate-and-fire (LIF), and Hodgkin-Huxley (HH) models. Among them, IF and LIF models describe the bio-plausible neuronal activity with a superficial explanation of the biophysical reason for electrical activity. A simple device or circuit with threshold properties is generally sufficient to achieve the IF and LIF neuron. On the other hand, the biophysical HH model precisely describes the ion dynamics at the ion channels during the spiking (Yang et al., 2019a). Although this model presents some advantages over the IF and LIF such as neuron-like precision control of the spiking rate, its realization generally demands very complex circuits. Up-to-date developments of 2D materials-based artificial neurons can be classified into two categories: some reports demonstrated artificial neurons with 2D materials-based TSMs (Chen et al., 2019b; Dev et al., 2020; Hao et al., 2020; Kalita et al., 2019), whereas others employed 2D materials-based FETs (Bao et al., 2019; Beck et al., 2020; Das et al., 2019; Hu et al., 2017).

#### 2D TSM-Based Neurons

**Figure 9:**
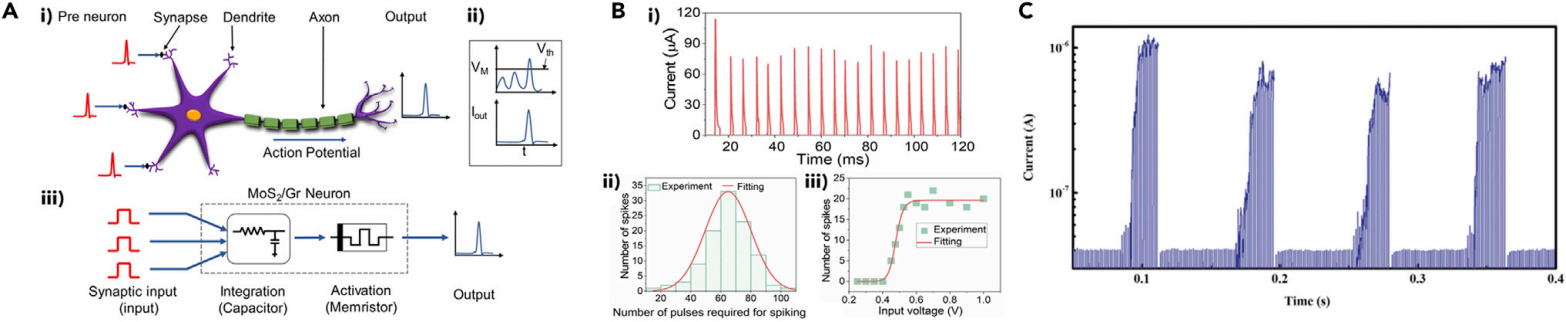
2D TSM-Based Neurons (A) (i) Schematic representation of a biological neuron from pre-neuron to output. (ii) Output spiking of neuron with respect to the input threshold. (iii) Conceptual representation of a MoS_2_/graphene memristor-based artificial neuron. Reprinted with permission from (Kalita et al., 2019). Copyright 2019 Springer Nature. (B) (i) Continuous output spikes of LIF neuron realized with a memristor device integrated with the RC circuit. (ii) The normal distribution of variation in the number of pulses required for generating an output spike. (iii) The variation of the number of output spikes in a given time period with increasing input pulse amplitude. The variation follows the sigmoid function. Reprinted with permission from (Dev et al., 2020). Copyright 2020 IEEE. (C) Output spikes of LIF neuron realized with a single memristor device. Reprinted with permission from (Hao et al., 2020). Copyright 2020 WILEY-VCH Verlag GmbH & Co. KGaA, Weinheim.

Kalita et al. adopted a vdW heterojunction TSM device composed of CVD-grown multilayer 2D MoS_2_ and graphene in accomplishing an artificial neuron (Kalita et al., 2019). The device exhibited a threshold voltage (V_th_) of ∼2.8–4.2 V and showed consistent volatile switching characteristics up to 1 μA. It was postulated that the grain boundaries-mediated charge transport within polycrystalline MoS_2_ was responsible for the threshold switching. The device was integrated with a resistor-capacitor (RC) circuit to demonstrate an artificial neuron, as shown in Figure 9A (Kalita et al., 2019). Upon application of input voltage pulses, the RC circuit integrated the incoming signals and built up the potential across the TSM device. Accordingly, the device initially at a high-resistance state (HRS) switched to a low-resistance state (LRS) as soon as the potential across it reached the V_th_. The LRS state of the device enabled the capacitor to discharge through the load resistor and caused a reduction of the voltage across it, hence the device reverted to HRS. The consecutive and reversible switching between HRS to LRS generated an output voltage spike. The authors compared the process of generating output spikes with the all-or-nothing and threshold-driven properties of LIF neurons. The work also showed that the frequency of output spikes is dependent on the amplitude of the input voltage pulse, which emulates the strength-modulated frequency response of the biological neuron. Even though the device demonstrated the basic properties of an artificial neuron, it suffered from high threshold voltage, low ON-OFF ratio, and low ON current, leaving its integration with a synaptic circuit and energy-efficient operation challenging. To improve these parameters, Dev et al. and Hao et al. independently incorporated Ag electrodes into MoS_2_-based TSM devices (Dev et al., 2020; Hao et al., 2020). Both reports showed that the positive voltage applied at the Ag electrodes caused the Ag^+^ to diffuse through the MoS_2_ to form conductive filaments, hence making HRS-to-LRS transition. The vertical 2D memristive structure of Au/MoS_2_/Ag demonstrated by Dev et al. also showed threshold switching characteristics with a low threshold voltage of ∼0.35–0.4 V and a high ON-OFF ratio of 10^6^. This work used an external RC circuit and TSM device to emulate the integration process of membrane potential and ion dynamics of biological neurons, respectively. The artificial neuron of the Au/MoS_2_/Ag TSM device emulated all the fundamental features of biological neurons with considerably lower input voltage pulses compared with the previous reports. Furthermore, the statistical study showed that the stochastic distribution of the number of pulses required for generating output spike followed a normal distribution. The variation of the number of output spikes achieved with the input pulses followed the sigmoid function, a widely used activation function for SNN as shown in Figure 9B (Dev et al., 2020). Consequently, this artificial neuron offered flexibility to modulate the number of output spikes by tuning circuit parameters, including resistance, capacitance, and RC time constant. Hao et al. optimized the channel length of monolayer MoS_2_ (500 nm) in a lateral TSM device to acquire the volatile behavior. The device showed a threshold voltage of ∼1.2 V and an ON/OFF ratio of ∼10^4^. Consistent volatile properties were achieved with a compliance current of 1 μA, whereas it was lost with an increase in the current value. The artificial neuron demonstrated in this work did not incorporate an external circuit, and the device connected with the pulsing voltage source initially did not respond to the pulse train with a voltage amplitude lower than V_th_. After a certain time frame similar to the integration period of a biological neuron, the device turned ON, which resembled the firing of the biological neuron, as shown in Figure 9C (Hao et al., 2020). The integration period was tunable with the amplitude of the incoming pulse train. The device recovered from the firing state with an appropriate time interval (50 ms) between the set of pulse trains, which is analogous to the leaky behavior of biological neuron. A memristive ANN was fabricated by incorporating MoS_2_-based neuron and Cu/GeTe-based synapse to demonstrate the computing ability of the neuron.

Chen et al. made another demonstration of LIF neuron without an external circuit by using a 2D MXene (Ti_3_C_2_)-based TSM device (Chen et al., 2019b). The Cu/MXene/Cu memristor device showed bidirectional volatile switching with a threshold voltage of 0.68 V. The OFF-state current was high (∼10 μA), and it lost the volatile characteristics only after 3 consecutive cycles. The conductance of the device at HRS was explained by the space-charge-limited conduction mechanism, whereas the resistive switching was attributed to the formation of Cu filaments through the MXene. The positive pulse train with constant amplitude caused a gradual increase in the device current, and the current increased sharply after a certain number of pulses. This behavior was compared with the integration and firing characteristics of a biological neuron. The number of pulses required for the sharp increase of current was higher for pulse train with the higher pulse interval, which indicated the leaky nature of the artificial neuron. The recovery time was around 0.6 s after the neuron firing. For SNN, TSM has been found to be more viable because its volatile characteristics eliminate the need for an external RESET circuit.

#### 2D FET-Based Neurons

**Figure 10:**
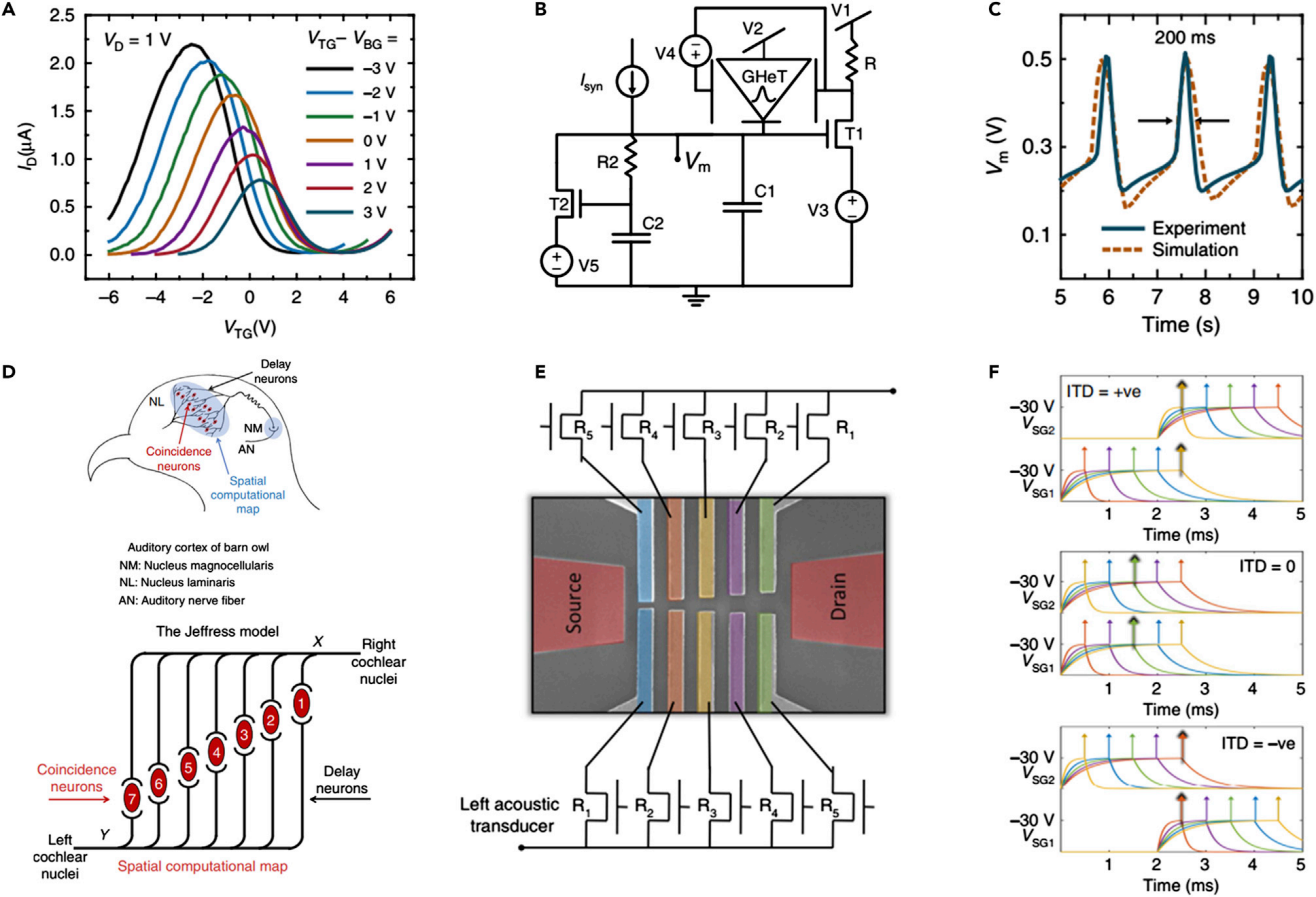
2D FET-Based Neurons (A) Dependent gate operation of GHeT. (B) Circuit diagram of a spiking neuron realized with GHeT. (C) Experimental and simulated output spikes of a GHeT-based neuron. Reprinted with permission from (Beck et al., 2020). Copyright 2020 Springer Nature. (D) An anatomical drawing of barn owl's brainstem, which shares remarkable similarity with the Jeffress model. (E) Fully integrated biomimetic audiomorphic architecture. (F) The way of charging of the split gate with the acoustic transducers at the two ends of the chip receiving the sound signal with positive, zero, and negative interaural delays. Reprinted with permission from (Das et al., 2019). Copyright 2019 Springer Nature.

FET-based approaches employing 2D materials have been explored to realize artificial neurons (Bao et al., 2019; Beck et al., 2020; Das et al., 2019; Hu et al., 2017). Recent reports demonstrated the viability of FET-based neurons for applications in sound localization (Das et al., 2019) and logic gate operation, along with single-neuron implementations. Beck et al. demonstrated a spiking neuron operational by the HH model adopting a Gaussian heterojunction transistor (GHeT) (Beck et al., 2020). CVD-grown monolayer MoS_2_ and carbon nanotube (CNT) were used to form the p-n junction for dual-gated GHeT. The drain current showed an anti-ambipolar response under the dual-gate control, as shown in Figure 10A. The height, peak position, and full width at half maximum (FWHM) of the anti-ambipolar response can be tuned by varying the offset between the top and bottom gate voltages. The tunable anti-ambipolar response of the GHeT was availed to demonstrate the spiking neuron. The circuit incorporated GHeT, n-type FET, resistors, and capacitors to emulate the Na^+^ channel of a biological neuron, whereas the K^+^ channel was imitated by delayed turn ON of an n-type FET (Figure 10B). Depending on the input synaptic current (I_syn_), OFF current (I_OFF_), and peak current (I_peak_), the GHeT turned ON and OFF sequentially and generated the continuous spikes at the output terminal as shown in Figure 10C. The simulation data, coupled with the experimental demonstration, showed that the spiking neuron consumed energy at ∼250 nJ per spike. Furthermore, they depicted that the circuit can generate constant spiking, class-I spiking, phasic spiking, phasic bursting, and tonic bursting by varying the biasing at the top and bottom gates.

An additional noteworthy demonstration of the coincidence neuron for audiomorphic computing application was reported by Das et al. using an exfoliated MoS_2_-based split-gate transistor (Das et al., 2019). Accurate sound source localization by identifying the interaural time difference (ITD) as in biological creatures is a complex computational task. The Jeffress model, which closely resembles the brainstem of barn owls, explains how the nerve cells process the acoustic timing difference to determine the source of a sound. The three main neural components of the Jeffress model are the time delay neuron, the coincidence detector neuron, and the spatial computational map (Figure 10D). The concurrent arrival of spikes at the corresponding delay neuron only turns on the coincidence neuron. The distance of the delay neuron from the right and left cochlea is maintained in such a way that only one coincidence neuron fires at a time, so that the source of sound can be precisely localized. Das et al. fabricated a MoS_2_-based multiple split-gate transistor, which turns on only when a pair of split gates receive the input voltage pulse simultaneously. Electrical measurements showed the maximum value of the ON/OFF ratio, termed as the inhibition ratio, whereas voltages were applied to vertically aligned split gates. This behavior was applied to mimic the coincidence neuron. The delay neuron was realized with a full top-gated MoS_2_ FET, where the delay or time constant was controlled by the top gate voltage. To build complete biomimetic audiomorphic architecture, the drain terminal of delay neurons was connected to each split gate of the coincidence neuron, as shown in Figure 10E. By varying the channel width and length, the resistance of the delay neuron was monotonically increased right-to-left and left-to-right for the top and bottom gates, respectively. With this arrangement, three different vertically aligned split pair gates of coincidence neurons received the coincident signal for positive, negative, and zero ITD (Figure 10F). The ability to tune the current of the coincidence neurons with the back gate accorded the feature of neuroplasticity to the audiomorphic device.

Bao et al. demonstrated a dual-gate exfoliated MoS_2_-based neuristor (Bao et al., 2019). A PEO:LiClO_4_ ionic gate was used as the top gate dielectric, which controlled the ionic migration, whereas dielectric SiO_2_ controlled the electronic migration as the back gate. Employing the ionic migration of Li^+^ from the top gate, synaptic characteristics, including STP, LTP, and PPF, were clearly demonstrated. In this neuristor, both top and back gates were utilized to emulate the propagation of action potential through the axon of biological neurons. The sampling clock applied at the back gate was not capable of populating the MoS_2_ channel with n-type carriers, hence no current flowed from the drain to source. In this condition, the drain terminal voltage remained high compared with the source terminal. Once the top gate was fed with input voltage pulses at the same frequency of sampling clock at the back gate, the device turned ON, and the potential at the source terminal increased to drain potential similar to the action potential propagation at axon. The variation of output spikes was dependent on the amplitude of the input voltage pulse at the top gate and followed the sigmoid function. The simulation using the shifted and scaled sigmoid function obtained from the neuristor resulted in faulty output at neural networks for written digit recognition. However, the performance can be improved by the parameter optimization of the algorithm.

Hu et al. reported an exfoliated MoS_2_-based coplanar neuron transistor with one floating gate and two control gates (Hu et al., 2017). The ability to control the transistor with both control gates simultaneously was implemented to emulate the summation function of a biological neuron. To demonstrate the neuromorphic application, abacus-like counting scheme, AND logic, and OR logic were established using this transistor. For logic gate application, two control gates were fed with square waves at different frequencies, and the drain current level was labeled as high/low state. These reports imply that 2D materials possess promising potentials for hardware-based neural networks.

## Challenges and Outlook

In summary, we reviewed the intrinsic properties of 2D materials and their heterostructures that make them advantageous for futuristic brain-like neuromorphic computing based on artificial synapses. There have been multiple studies on the applications of these 2D materials via different mechanisms that show unprecedented opportunities for achieving 2D-based neuromorphic devices. In addition, we offered an extensive overview of various 2D and 2D heterolayers-based neuromorphic devices in terms of their growth and fabrication methods, operation mechanisms, and synaptic properties. Although 2D materials offer immense advantages compared with conventional materials, there are still several challenges that hinder the implementation of 2D materials for brain-like computing devices in real-world applications. The future technological advancement and proliferation of 2D materials-based neuromorphic applications entail holistic cooperative approaches across diverse disciplines. These embrace large-area defect-free material preparation and manufacturing, low-footprint hetero-integration into devices achieving reliable performances, as well as high energy efficiency and high-density integration in terms of system-wide metrics. From the foundational material-level perspective, there remain major limitations in the large-scale growth of high-quality 2D materials films with uniform and controllable thickness, which is imperative in achieving high-density 2D-based neuromorphic networks for brain-like computing. Unlike the single-element graphene whose scalable CVD growth is industrially compatible at present (Li et al., 2009), matured techniques to grow compound 2D materials such as TMDs accompanying controlled morphology and industrial scalability still remain unavailable. With these endeavors, Chen et al. synthesized millimeter-scale MoS_2_ by reducing nucleation sites (Chen et al., 2017a) and Yang et al. obtained a 6-inch monolayer film of polycrystalline MoS_2_ by utilizing molybdenum foil as a precursor (Yang et al., 2018). However, it still remains challenging to precisely control the microstructure of TMDs, such as thickness, defects, and doping, all of which are critical parameters to determine their quality and properties. Furthermore, practical implementations call for the development of wafer-scale techniques for deterministically transferring high-quality as-grown 2D materials onto arbitrary substrates to guarantee mechanically deformable 2D-based neuromorphic computing devices. The currently available techniques present intrinsic limitations in terms of enabling the transferred 2D materials to preserve their as-grown quality. Particularly, one of the most significant challenges lies in how to retain the atomically sharp and clean interfaces among heterogeneously stacked distinct 2D layers avoiding structural and chemical inhomogeneity in between them. Various approaches have demonstrated the feasibility of the wafer-scale 2D layer transfer; these include polymer-assisted layer-by-layer transfer, strain-driven mechanical cracking, and water-assisted direct integration of 2D layers (Han et al., 2020; Kang et al., 2015; Kim et al., 2019a; Shim et al., 2018; Wang et al., 2020). However, it still remains technically challenging to congruently accompany wafer-level scalability and atomic-level precision in transferred 2D layers to ensure the reliable operation of complicated neural networks in a highly predictable manner. With respect to the challenges associated with device fabrications, considerable efforts are still demanded in achieving small-footprint vdW patterning and integration without compromising intrinsic switching performance and neuromorphic functionality. Particularly, one major hurdle is the unreliable stochastic nature of operating characteristics inherent to 2D materials-based neuromorphic operations. Most of the up-to-date devices exhibited significant batch-to-batch and cycle-to-cycle variations of essential parameters such as SET/RESET voltages, operation current, and data retention, which become more pronounced with repeating operations. For example, even though the grain boundaries-mediated operation of memtransistors was demonstrated with monolayer MoS_2_, this switching principle is intrinsically limited and practically unscalable due to the spatial inhomogeneity of CVD-grown grain boundaries (Sangwan et al., 2015). Similarly, despite the intrinsic synaptic behavior of multilayer h-BN as a resistive switching medium, it is difficult to control the locations and dimensions of conductive nanofilaments formation within them (Shi et al., 2018). Furthermore, the resistive switching devices composed of monolayer TMDs in between metal electrodes often exhibit limited performances such as low endurance (Ge et al., 2018). The instability of these 2D memristors is attributed to multiple factors, including the progressive degradation of properties in 2D materials as well as their uncontrolled structure and chemistry. Furthermore, the fundamental principles responsible for the intrinsic electrical switching in 2D materials still remain largely unclarified. Specifically, underlying factors that govern the spatial randomness associated with the formation and annihilation of the conductive pathways within switching media should be identified. Consequently, viable methods for their well-defined spatial control should be established to improve the reliability of the devices, which should be extended to the system-level implementation and architectures. All these efforts should be holistically driven in every aspect of development, i.e., material design, device fabrication, and circuit integration, to leverage the present proof-of-concept demonstrations toward the practically relevant brain-like computing systems in real-world applications.
